# Sensitive, direct detection of non-coding off-target base editor unwinding and editing in primary cells

**DOI:** 10.1101/2025.09.25.678665

**Published:** 2025-09-25

**Authors:** Tong Wang, Selin Jessa, Georgi K. Marinov, Sandy Klemm, Anshul Kundaje, William J. Greenleaf

**Affiliations:** 1 Department of Pathology, Stanford University, Stanford, CA, USA; 2 Department of Genetics, Stanford University, Stanford, CA, USA; 3 Department of Applied Physics, Stanford University, Stanford, CA, USA; 4 Department of Computer Science, Stanford University, Stanford, CA, USA

## Abstract

Base editors create precise nucleotide changes in DNA, but their off-target activity remains challenging to quantify. Here, we develop and deploy a direct, *in cellulo* sequencing assay that simultaneously measures both Cas9-mediated unwinding and deaminase editing of genomic DNA (beCasKAS). Our strategy nominates >460-fold more potential off-target sites than other methods by enriching for Cas9-dependent R-loops immediately preceding editing. Using beCasKAS in primary human T-cells, we observe that mRNA-encoded ABE8e and PAMless ABE8e-SpRY base editors have distinct off-target profiles that can be mitigated by optimizing mRNA dose. Finally, we combine beCasKAS with base-resolution deep learning models to risk-stratify off-target edits by their likelihood of epigenetic dysregulation. Collectively, beCasKAS offers a sensitive and facile tool to optimize the balance between base editor on- and off-target activity.

CRISPR base editors are promising genetic editing technologies that underlie at least 19 active clinical trials globally (1). Despite the enormous potential of base editing, the impact of off-target activity remains challenging to comprehensively quantify, both because identification of potentially rare edits at off-target sites often requires complex molecular assays of uncertain sensitivity, and because off-target edits impacting non-coding regions are difficult to classify as detrimental or benign. Currently no existing strategy can detect base editor specific off-target effects within primary cells, which would allow a human gene therapy product to meet Food and Drug Administration's (FDA) ideal recommendations (2). Many off-target detection methods are based on *in silico* nomination (3, 4) or *in vitro* biochemical assays (5-8), but these approaches generate many false positives and negatives. While *in cellulo* methods exist for detecting off-target base edits, they have not been applied in primary cells likely due to several limitations: these methods optimally detect double-stranded breaks (9-12), can only be used on one type of base editor (13, 14), or require whole-genome sequencing (15, 16). As a result, controversy remains on whether off-target base edits are non-detectable, random, or systematically enriched at sites of Cas9 guide RNA (gRNA) binding (13, 15, 16).

To address the critical need for sensitive detection of CRISPR off-targets, we previously reported CasKAS (**Cas K**ethoxal-**A**ssisted **S**ingle-stranded DNA sequencing), a rapid, inexpensive assay that directly detects DNA editor R-loops genome-wide both *in vitro* and *in cellulo* using the commercially available N_3_-kethoxal reagent (17). Although CasKAS can be used to discover the off-target sites made by any ssDNA forming genome editor, we reasoned it would be especially useful for characterizing base editors (Fig. 1A). In most base editors, a C>T or A>G edit can be site-specifically installed by a deaminase within a small single-stranded DNA (ssDNA) editing window created by the Cas9 R-loop (Fig. 1B) (18, 19). The base editing deaminases are intentionally chosen to have only ssDNA and not dsDNA activity to limit off-target toxicity. Furthermore, Cas9 R-loop formation has also been shown to be rate-limiting in biophysical studies (20). We therefore posited that an assay detecting R-loop formation, rather than binding, may be especially useful for base editors employing ssDNA deaminases (21, 22). In our base-editor CasKAS (beCasKAS) approach, we use an N_3_-kethoxal reagent to label unwound R-loops, pull these regions down, and use sequencing to detect off-target deaminase edits (Fig. 1C) (23).

Here, we develop and deploy beCasKAS in both cell lines and primary human T cells. Using beCasKAS we find a wide editing window for the highly active editor eBE and a narrow editing window for the extensively evolved ABE8e (24, 25). We measure deaminase-specific mutational sequence contexts intrinsic to these enzymes and uncover a minimally homologous 5-nucleotide PAM-adjacent seed for unwound R-loops. Surprisingly, we estimate the frequency of gRNA-dependent ABE8e edits in HEK293T cells to be only ~1.9-fold less than for eBE, although eBE generates ~20-fold more edits per R-loop than ABE8e. We additionally optimize a primary human T cell compatible beCasKAS workflow and demonstrate a means to quantitatively measure off-targets across increasing concentrations of base editor enzyme, allowing for identification of optimal editor dose for therapeutically relevant clinical targets. Finally, we use a deep learning approach to triage off-target non-coding sites found by beCasKAS predicted to affect DNA accessibility in primary human T-cells.

## Results:

### beCasKAS directly detects unwinding and editing in cellulo

We selected the cytosine base editor eBE and adenine base editor ABE8e as two contrasting DNA editors to evaluate by beCasKAS (24, 25). While these editors have never been directly compared experimentally, eBE employs the endogenous human deaminase APOBEC3A (hA3A) which exhibits significant off-target activity compared to other cytosine deaminases and also is known to be dysregulated in multiple human cancers (26, 27). In contrast, ABE8e is a widely used adenine base editor thought to exhibit considerably less off-target activity (25). We used the previously studied HEK4 gRNA which allowed us to benchmark against other existing *in cellulo* off-target detection methods (9, 11, 13).

**Fig. 1:**
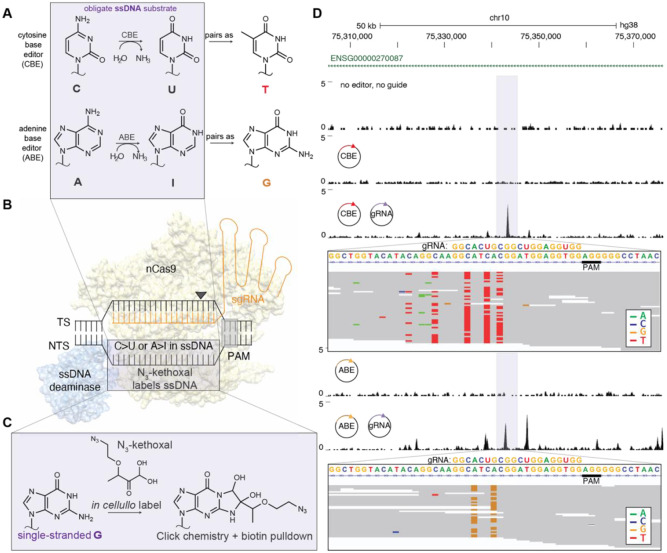
beCasKAS directly detects unwinding and editing *in cellulo.* **A)** Biochemical reactions of CBEs and ABEs. Most CBE and ABE editors require ssDNA substrates. **B)** Cartoon representation of the R-loop intermediate in canonical CBEs and ABEs. CBEs have an additional DNA repair inhibitor that is not shown. **C)** N_3_-kethoxal reagent covalently labels ssDNA at unpaired guanine nucleotides. **D)** Representative genomic track showing a gRNA dependent off-target R-loop. Sequencing reads within the highlighted peak (purple) show C>T edits (red) and G>A edits (green) for the CBE. A>G edits (gold) are seen for the ABE. The targeting gRNA and PAM are shown.

We transfected base editor and gRNA plasmids into HEK293T cells. After one, two, and three days of editing, cells were treated with the cell-permeable N_3_-kethoxal reagent to covalently label ssDNA. After gDNA extraction, biotin labels were attached to the ssDNA using a click reaction, DNA was sheared, and a streptavidin pulldown was performed before library preparation (see Methods). One day after transfection, gRNA dependent Cas9 R-loops were detected at a highly homologous off-target site (Fig. 1D, purple). At this site, for the CBE, visible C>T (red) and G>A (green, opposite strand C>T) editing was observed near the PAM (gray) distal end of the gRNA sequence. In contrast, only A>G (yellow) edits were observed for the ABE enzyme. Thus, our approach enables detection of both unwound gDNA in R-loops (as gRNA-dependent beCasKAS peaks), as well as individual base edits (as single nucleotide variants in beCasKAS reads).

### beCasKAS quantifies strand-specific behavior of base editors genome-wide

**Fig. 2:**
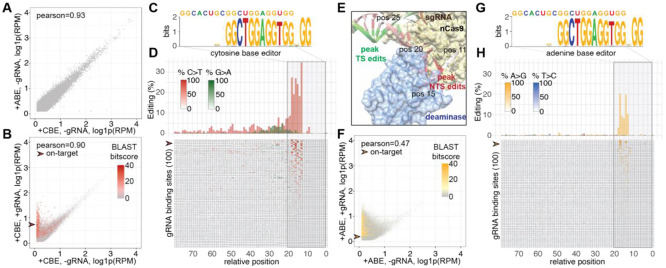
beCasKAS validates known properties of CBE and ABE at sites with significant homology. **A)** Contiguous, non-overlapping 1-kbp bins of genome-wide ssDNA signals detected in beCasKAS without gRNA. **B)** Contiguous, non-overlapping 1-kbp bins for CBE +/− gRNA conditions. Overlaid is the BLAST bitscore. The on-target peak is highlighted with the arrowhead. **C)** All CBE BLAST hits identified after multiple sequence alignment as a WebLogo. **D)** The top 100 most edited BLAST identified regions for CBE are shown as a bar-graph, and each individual site is shown below in heatmap format. Distances are relative to the PAM. The on-target peak is highlighted with the arrowhead. **E)** Editing frequency of cytosine base editor overlaid on a crystal structure of a base editor in complex with DNA (PDB: 6VPC). The numbers correspond to relative position on the gRNA non-target strand (NTS) as in panel **D)**. **F)** Contiguous, non-overlapping 1-kbp bins of ssDNA signals in beCasKAS for ABE +/− gRNA conditions. **G)** All ABE BLAST hits identified after multiple sequence alignment as a WebLogo. **H)** Top-100 most edited BLAST identified regions for ABE.

To assess native genome-wide beCasKAS patterns, we measured beCasKAS signal over contiguous, non-overlapping 1-kbp bins after transfection with either ABE or CBE but no gRNA (Fig. 2A). We observed similar ssDNA signals in these two conditions (Pearson’s r = 0.93), although we found four regions which appeared significantly enriched in the CBE-only over ABE-only conditions (Fig. S1A). These reads mapped to exons in the hA3A gene with mismatched bases over intronic regions, suggesting that beCasKAS was detecting transcriptionally active ssDNA from the transfected plasmid and not genomic DNA. We also evaluated the ssDNA landscape with or without the presence of gRNA for the CBE (Fig. 2B). We found that the CBE generated many putative gRNA-dependent R-loops (Pearson’s r = 0.90), and observed strong correlations between beCasKAS replicates (Fig. S1B) performed on the same (Pearson’s r = 0.91) and independent days (Pearson’s r = 0.89).

We next sought to validate highly homologous gRNA off-target sites. We called sharp peaks enriched in pulldown relative to input conditions using MACS2 (henceforth labelled “total peak calls”) and focused on the day 2 time point, which showed significant editing and unwinding of off-target sites (Fig. S1C) (28). Following the strategy of previous off-target identification efforts, we used BLAST to determine if any gRNA-homologous regions could be found within the called peaks (9, 29). We found 547 BLAST hits with NGG seeds for the CBE. Among the hits with significant BLAST homology, we further generated a position weight matrix (PWM) to understand the determinants of gRNA-dependent off-target binding (Fig 2C). We found the PAM-adjacent and not PAM-distal bases to be most important, consistent with the existing model for seed-dependent Cas9 interrogation of genomic DNA (30, 31).

We next visualized the top 100 most edited BLAST hits for the CBE and plotted their average conversion frequency as a function of distance from the PAM (Fig. 2D). The on-target site, which was the 4th most edited site overall, showed 68.3%, 78.3%, and 64.4% editing at three target cytosines in positions −18, −16, and −13 relative to the NGG PAM. Across all 100 sites, we found that C>T edits predominantly occur ~13-19 bases upstream of the PAM, consistent with the intended base editor behavior of converting bases within the editing window of the gRNA non-target strand (NTS). However, NTS edits are also detectable significantly outside of the protospacer, consistent with prior reports using other base editors (13, 32).

We also observed frequent gRNA target strand (TS) edits (G>A changes on the positive strand) that peaked 20-30 bases upstream of the PAM (green), staggered from the C>T edits. Overlaying these editing frequencies on a crystal structure of a base editor in complex with DNA (Fig. 2E) (33) provided structural justification of these patterns, as the NTS is most proximal to the deaminase 10-20 bases upstream of the PAM, while the TS is most proximal to the deaminase ~25 bases upstream of the PAM. We additionally found slight ~10-11 nucleotide periodicity of C>T edits on the NTS with local maximums occurring at 38, 49, and 60 nucleotides 5’ to the PAM. These findings suggest that the helical turn of DNA affects editing efficiency for out-of-protospacer edits for eBE, and that more broadly, beCasKAS can detect intrinsic strand-specific preferences of base editors.

We repeated this analysis to interrogate genome-wide patterns of beCasKAS applied to ABEs. In contrast to the CBE editor, the ABE dramatically distorted the endogenous ssDNA landscape (Pearson’s r = 0.47. Fig. 2F). Using BLAST, we found 521 candidate off-target sites at the day 2 time-point that were enriched for the PAM-adjacent seed sequence (Fig. 2G). ABE8e does not show any out-of-protospacer or TS edits (blue) and instead only has significant activity in the known target window 13-18 nucleotides upstream of the PAM (Fig 2H) (25). The on-target site is also the 4th most edited site, with target A edited at 70%. Taken together, beCasKAS identifies high-probability gRNA-dependent off-target R-loops as these regions recapitulate known, distinct properties of the CBE and ABE tested.

### beCasKAS detects off-target, base-edited R-loops with limited gRNA homology

**Fig. 3:**
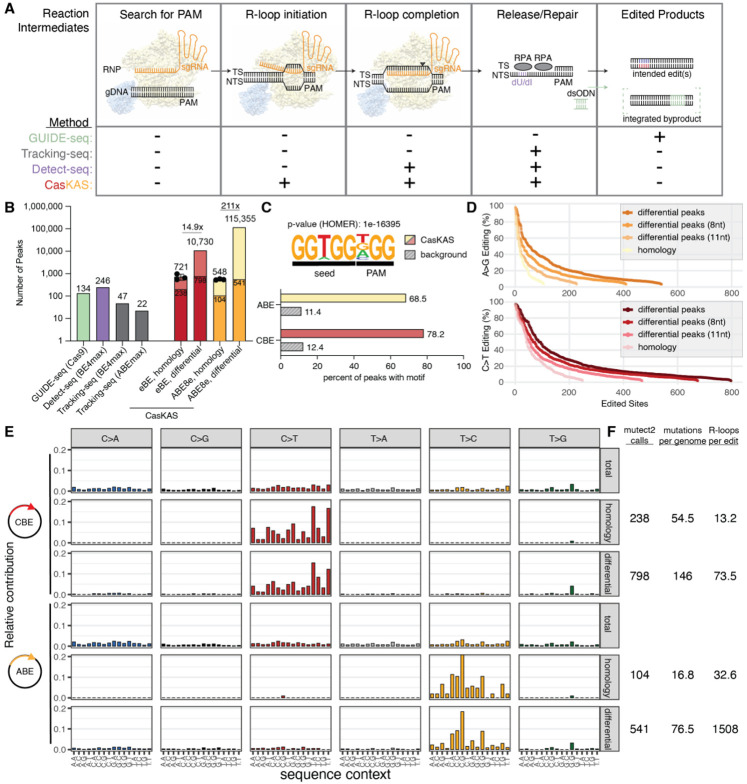
beCasKAS has a highly sensitive, distinct mechanism of enrichment. **A)** Reaction intermediates for base editing. beCasKAS directly measures gRNA-dependent R-loops which occur earlier in time compared to intermediates used by other off-target identification methods. GUIDE-seq measures integration of a transfected oligo (dsODN) at sites with WT Cas9 induced DNA breaks (11). Tracking-seq indirectly measures the presence of Replication Protein A (RPA) (9). Detect-seq measures deoxyuracil (dU) intermediates generated only by cytosine base editors (13). **B)** Comparison of beCasKAS to other *in cellulo* methodologies. beCasKAS R-loops can be found in two ways: homology (BLAST) or differential peaks in BE +/− gRNA conditions (DESeq2 FDR < 0.05 for n=3 conditions). The darker color shows edited peaks. **C)** HOMER *de novo* motif discovery for peaks occurring in differential (DESeq2) peaks versus GC-content matched background. **D)** Rank ordered edited sites identified by differential peaks (DESeq2) and homology (BLAST). “8nt” and “11nt” lines represent R-loops with seed matches of at least that length. **E)** Mutational profile of variant calls. **F)** Absolute editing efficiencies and their relationship to unwound ssDNA peaks for CBE and ABE.

Although BLAST identifies highly homologous sites enriched by beCasKAS (721 for CBE, 548 for ABE), we observed a large number of regions genome-wide that were not found by BLAST but had differential read counts with and without gRNA (Fig. 2B/F), suggesting that beCasKAS may be identifying *bona fide* off-target sites missed by other approaches (Fig. 3A). To investigate R-loops not identified by BLAST’s homology-directed sequence search (a strategy henceforth labelled “homology” peak calling), we employed a DESeq2-based workflow to identify consensus differentially identified peaks in the +/− gRNA conditions with an FDR of <0.05 (Fig. S1D, a strategy henceforth labelled “differential” peak calling) (34). We found that the CBE and ABE enzymes generate 14.9x and 211x, respectively, more differential peaks than found by homology matching for the gRNA sequence (Fig. 3B). This approach nominates 469x more putative off-target sites than the next most sensitive *in cellulo* base editor method (115,355 beCasKAS vs 246 Detect-Seq = 469x), consistent with how beCasKAS enriches for more reaction intermediates preceding uracil generation (13). Of the CBE differential peaks, 96.3% were also called in the ABE condition, suggesting these regions are strongly Cas9-dependent (Fig. S1E). In contrast, 91.0% of the gRNA-dependent peaks for the ABE were not identified in the CBE dataset. Because equal amounts of plasmid DNA with the same CMV promoter driving editor expression was delivered for each condition, we speculate that the difference in off-target sites detected in the ABE relative to CBE is likely explained by the ABE’s codon optimization and smaller size (lack of C-terminal uracil DNA glycosylase inhibitor fusion) relative to the CBE.

To understand the sequence determinants of the off-target differential peaks, we performed a *de novo* motif search using HOMER (Fig. 3C) (35), identifying a 5-nt seed + 3-nt PAM as the top hit for the HEK4 gRNA (p-value: 1e-16395). This motif occurs in 68.5% of putative ABE R-loops, and 78.2% of CBE R-loops while it only occurs in 11.4% or 12.4% of the GC-content matched genomic background, respectively. Thus, gRNA-dependent R-loops are enriched for a 5-nt PAM adjacent seed. Consistent with expectation, we also found that as gRNA seed homology decreases, more off-target sites with detectable base edits are found (Fig. 3D).

To understand the effect of local sequence context on editing efficiency, we next characterized the mutational patterns of both editors. Within the base editor protein, the deaminase enzyme that is chosen contains an evolutionarily divergent recognition loop which confers local sequence context biases to editing efficiency (36, 37). We find that total peak calls (by MACS2) exhibited no mutational sequence preferences, suggesting that the deaminase editing signature is undetectable within regions of genome-wide ssDNA under these assay conditions (Fig. 3E). In contrast, CBE peaks with BLAST homology showed a strong C>T mutational pattern and enrichment of the 5’TC sequence context, the sequence context known to be preferred by the hA3A deaminase (38). The 798 total C>T variants in differential peaks also shared the same mutational pattern as the 238 variants observed in homology-based peak identification, suggesting these variants are *bona fide* base editor off-target sites, despite their limited gRNA homology. We also uncovered a consistent ABE8e sequence context mutational pattern for the 104 T>C variants (opposite strand A>G) called in homology peaks and 541 total T>C variants in differential peaks.

We employed amplicon sequencing to investigate how editing within an R-loop is related to absolute editing frequency. We found a linear relationship for both base editors, where the observed editing within the amplicon is 84% and 66% of the editing within the beCasKAS detected R-loop for the CBE and ABE, respectively (Fig. S1F). Using these values as the occupancy of the base editor at every site, we can estimate an absolute mutation frequency of 146 eBE edits per genome and 76.5 ABE8e edits per genome within gRNA dependent R-loops (Fig. 3F). Thus, eBE and ABE8e generate 73.5 and 1,508 peaks per edit, respectively, suggesting that the laboratory-evolved ABE8e deaminase has lower overall ssDNA editing efficiency per R-loop generated than the highly active eBE. Taken together, beCasKAS unearths surprisingly similar absolute mutation frequencies for the promiscuous eBE and the widely deployed ABE8e (146 / 76.5 = 1.91-fold) under these transfection conditions.

### Optimized beCasKAS in primary human T cells

Base editors have recently been used to *ex vivo* engineer allogeneic CAR-T cells for human patients. In one approach, a CD7-targeting CAR-T cell is generated by the simultaneous editing of *TRBC1* and *TRBC2*, *CD52*, and *CD7* to treat T-lymphoblastic leukemia (T-ALL) (39). Other genes can be targeted to create allogeneic CAR-T products including *PVR*, *CD3E*, *CIITA*, and *B2M* (40). We asked whether beCasKAS data could be used to engineer potentially safer allogeneic products, given these therapies have the potential for widespread clinical application beyond current autologous CAR-T therapies.

**Fig. 4:**
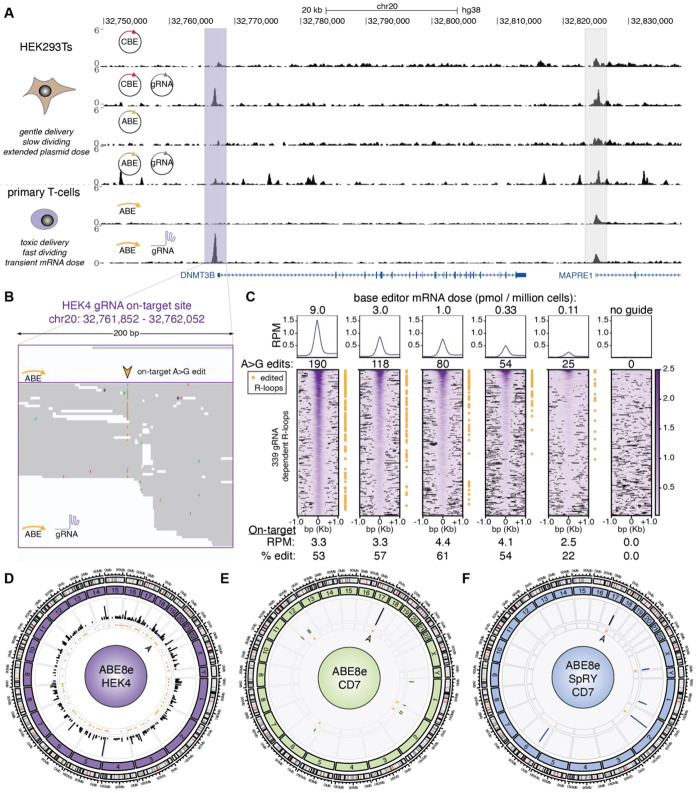
beCasKAS detects off-target sites in primary human cells. **A)** Browser tracks depicting HEK4 gRNA on-target site (purple) and nearby promoter (gray) in HEK293T and primary T cells. **B)** reads at on-target site with target A site marked (arrowhead). **C)** gRNA-dependent R-loops (heatmap) and edits (yellow dot) for the same guide and different base editor mRNA concentrations. **D)** ABE8e and HEK4 gRNA off-target R-loops (lines) and edits (yellow dots). **E)** ABE8e and CD7 gRNA off-targets. **F)** ABE8e-SpRY and CD7 gRNA off-targets. In panels **E)** and **F)**, shared off-target R-loops are shown with a black line while distinct off-targets are shown with colored lines. The arrowhead marks the on-target site in panels **D-F**.

We anticipated significant challenges for primary cell beCasKAS as no *in cellulo* base editor specific off-target method currently exists. First, base editor delivery in primary cells cannot be done with plasmids. We instead used 5-methoxyuracil (5moU) modified mRNA (Fig. S2A), a transient delivery method expected to result in fewer off-target edits than plasmid delivery (41). Second, activated T-cells divide rapidly, and any cells in S-phase will have exposed ssDNA which will randomly be labelled by our N_3_-kethoxal reagent (Fig. S2B). Finally, electroporation is toxic to T-cells, and extracellular DNA can also be labelled by N_3_-kethoxal. We therefore hypothesized that G1/S-blockade to limit replication, viability sorting, and extracellular nucleic acid degradation with DNase I could all be used to independently improve signal-to-noise in beCasKAS (Fig. S2C). By making these systematic modifications, we optimized the original beCasKAS protocol to improve the on-target signal by 11.1-fold (Fig. S2D). We next sought to compare primary beCasKAS on the same editor (ABE8e) and gRNA (HEK4) as used in HEK293T cells (Fig. 4A). Under these optimal conditions, the read depth-normalized on-target HEK4 gRNA site showed greater peak height relative to background in primary T cells than in HEK293T cells (purple, Fig. 4B).

We next aimed to understand whether base editor dose might affect off-target editing. We performed a dilution series with base editor mRNA, while keeping gRNA concentration and cell count constant. While on-target editing efficiency remained constant across a 27-fold dilution series (9.0 to 0.33 pmol mRNA per million T-cells), off-target R-loop formation decreased by 2.8-fold and off-targed editing decreased by 3.5-fold (Fig. 4C, Fig. S4). These results provide proof of principle that systematic mRNA dosing coupled with beCasKAS can provide on- and off-target unwinding and editing measurements that enable identification of an optimal therapeutic dose for base editing.

Although useful for benchmarking the performance of beCasKAS relative to other methods, the HEK4 gRNA is known to be more promiscuous than most therapeutic gRNAs (Fig. 4D). We thus identified off-targets for a splice donor-disrupting CD7 gRNA, which can be used in CD7-targeting CAR-T cells in order to escape fratricide (Fig. 4E) (39). In contrast to the 339 R-loops identified with the HEK4 gRNA, the CD7 gRNA identifies only 9 strong candidate R-loops, all with significant seed homology (>10 consecutive bases matching with the protospacer, Fig. S5A). Notably, we identified one off-target edit which creates a missense mutation in the oncogene/tumor suppressor protein NOTCH4 (T428A), with potential bystander edit (H430R), both of undetermined significance (Fig. S5B).

We additionally compared ABE8e to the more versatile, near-PAMless ABE8e-SpRY variant (Fig. 4F) (42). Although a PAMless enzyme might be expected to have many off-target sites, we wondered if this would be true using beCasKAS, which uniquely detects R-loop formation (Figure 3A). Across both beCasKAS experiments we found only one shared R-loop, the on-target CD7 peak (A>G edit: 76.3% for WT, 80.0% for SpRY). Interestingly, ABE8e-SpRY had four fewer off-target R-loops (four vs eight) and three fewer edited sites (two versus five) than ABE8e. We also compared R-loops discovered by beCasKAS for PVR/CD3E/CIITA/B2M quadruple edited T-cells. In this setting, the ABE8e-SpRY enzyme exhibited slightly lower editing efficiencies at the four target loci (Fig. S5C). The only shared on-target sites were the four on-target peaks, while we found five unique ABE8e off-target R-loops (three edited) and three unique ABE8e-SpRY off-target R-loops (two edited). Among the off-targets, we identify the known PVR gRNA off-target for the *ICAM-1* exon 2 splice donor, which has been shown to create protein knockdown of *ICAM-1* of uncertain therapeutic significance (Fig. S5D) (40). We additionally observed bystander edits which create missense mutations Y110H and W111R which both only occur in reads where the splice donor is also edited. Interestingly, despite having 17 out of 20 matches to the protospacer sequence, using the ABE8e-SpRY enzyme instead of the ABE8e WT enzyme dramatically reduces R-loop formation at this off-target site. Overall, beCasKAS demonstrates that 5moU-modified mRNA delivery of ABE8e or ABE8e-SpRY in primary human T-cells results in limited off-target edits for multiple therapeutic gRNAs. The limited number of R-loops detected for ABE8e-SpRY is also consistent with recent *in vitro* studies on SpRY-Cas9 describing how PAM-relaxed enzymes can be kinetically trapped after initial binding (43, 44).

### Base-resolution deep learning model predicts effects of non-coding off-targets

**Fig. 5:**
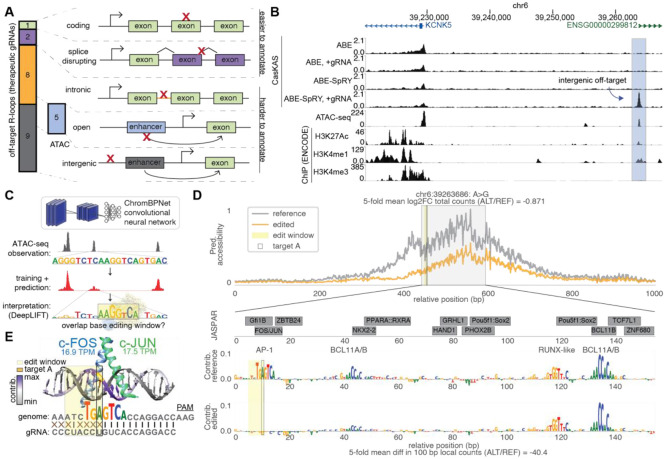
Base-resolution deep learning improves functional annotation of off-target sites overlying a cCRE. **A)** Locations of off-target R-loops for ABE8e and ABE8e-SpRY and five therapeutic gRNAs. **B)** Browser track of novel beCasKAS-detected ABE8e-SpRY + CD7 gRNA off-target site (blue) occurring in a difficult-to-annotate intergenic region, along with ATAC-seq data in primary T-cells generated in this study. The off-target site overlaps with ATAC-seq, H3K27Ac ChIP-seq (ENCODE accession ID ENCFF388LNF), and H3K4me1 ChIP-seq (ENCFF036KBQ), but not H3K4me3 ChIP-seq (ENCFF793LJI). **C)** Overview of deep-learning workflow. A ChromBPNet model is trained on ATAC-seq data to predict accessibility in 1,000 bp windows using 2,114 bp local DNA sequence as input. Models are interpreted by DeepLIFT to derive base-resolution scores representing contribution to accessibility. Editing windows from beCasKAS can be overlaid, with or without an observed mutation. **D)** First track: Predicted accessibility profile at off-target locus for chr6:39263686 A>G edit compared to the reference genome, using T-cell ChromBPNet model. Second track: JASPAR CORE 2024 motifs and base-resolution DeepLIFT contribution scores within the 150-bp ATAC-seq summit. Third track: Contribution scores of unedited (reference) sequence. Last track: Contribution scores after *in silico* chr6:39263686 A>G edit is performed. **E)** Crystal structure of c-Fos/c-Jun:DNA complex (PDB: 1FOS). The A>G edit within the editing window is colored in yellow and the target DNA strand has DeepLIFT contribution scores overlaid. Total RNA-seq quantifications of FOS and JUN expression in activated T-cells is also shown (in TPM units, Transcripts Per Million).

We next aimed to characterize the consequences of off-target edits for therapeutic gRNAs. Of the 20 off-target R-loops, only three were easily annotated as either missense or splice-disrupting (Fig. 5A). To map the overall *cis*-regulatory element (CRE) landscape and investigate if any off-target sites overlapped active CREs for the 17 intronic and intergenic off-target sites, we performed ATAC-seq in activated primary human T cells. Five sites were found to be in regions of accessible chromatin. Among these five sites, we discovered an ABE8e-SpRY and CD7 gRNA specific off-target site that overlapped with both an accessible region in our ATAC-seq dataset and ENCODE ChIP-seq peaks for the histone marks H3K27Ac and H3K4me1 in activated human T-cells (Fig. 5B).

Given that this off-target site could result in a disturbance of an active enhancer element in T-cells, we evaluated the effects of base edits using the ChromBPNet interpretable deep learning framework (Fig. 5C). ChromBPNet models learn to predict chromatin accessibility profiles from ATAC-seq data in 1 kb regions using only the local DNA sequence as input, while learning and correcting for Tn5 transposase bias (45). To identify the sequence features driving accessibility, we used the DeepLIFT algorithm (46) to interpret the model and extract base-resolution contribution scores in ATAC-seq peaks, representing the influence of each nucleotide on predicted accessibility (Fig. 5C). ChromBPNet has been used to predict the effects of sequence variants on chromatin accessibility, achieving correlations of 0.76 with accessibility effect sizes from molecular quantitative trait loci (QTL) studies, and correlations of 0.67 with expression effect sizes from CRISPR prime editor targeting screens (45, 47). Variants from genome wide association studies (GWAS) prioritized with ChromBPNet are also enriched for statistically fine-mapped variants with high posterior probabilities (45). We previously employed this framework to infer the sequence drivers of chromatin accessibility across 189 primary human cell types (48).

We first aimed to validate that ChromBPNet models could predict the known therapeutic effect of a well-characterized noncoding variant relevant to therapeutic CRISPR editing. We reasoned that our existing ChromBPNet model trained on human erythroblast ATAC-seq data (48) could simulate disruption of the *on-target* enhancer element targeted by the FDA-approved Casgevy (exagamglogene autotemcel). Casgevy is an autologous, *ex vivo* CRISPR-Cas9 gene therapy that disrupts the +58 intronic BCL11A erythroid enhancer in hematopoietic stem cells to restore fetal hemoglobin (HbF) expression for the treatment of sickle cell disease and beta thalassemia (Figure S6A) (49, 50). This +58 intronic BCL11A enhancer is accessible and active only in erythroid cells, and not in other cell types like the T-cells we characterized using ATAC-seq (Figure S6B). ChromBPNet accurately predicted accessibility at the BCL11A enhancers and contribution scores also revealed the known single GATA motif as the primary driver of accessibility at the +58 enhancer (Fig. S6C, middle track). While Casgevy employs CRISPR-Cas9 to disrupt the GATA motif, preclinical base editor studies similarly target this same motif to achieve the same therapeutic phenotype (51). We thus simulated the effects on accessibility of two T>C mutations within the ABE8e editing window, using the most optimal gRNA from this study. While one of the T>C edits show minimal effects on overall DNA accessibility (log2FC = −0.222, respectively), the intended on-target base edit results in nearly complete loss of predicted DNA accessibility (log2FC = −1.042) (Figure S6C-D). Base-resolution contribution scores confirmed that only the second T>C edit disrupted the GATA motif, underlying the predicted loss of accessibility (−47.67 change in local counts). Collectively, this experiment shows feasibility of using ChromBPNet to predict changes in accessibility associated with a well-established CRISPR therapeutic target in a non-coding regulatory region.

Given this promising result in the erythroid model, we trained new ChromBPNet models in a five-fold cross-validation scheme on our newly generated, donor-matched T-cell ATAC-seq data, and all models exhibited strong predictive performance (average Pearson’s r = 0.76; Fig. S7A-C, Table S3) For comparison, our prior datasets achieved an average Pearson’s r of 0.78 (IQR [0.75-0.81]; Fig. S7B) (48). The contribution scores highlighted four strong motifs corresponding to binding sites for AP-1, BCL11A/B, and RUNX-like transcription factors (TFs) spanning 27 of the 150 bp surrounding the ATAC-seq summit. In contrast, the JASPAR 2024 core database reports at least 13 different motifs spanning 110 of the 150 bp within this same region, demonstrating the difficulty of annotating variants without a cell-type specific deep learning model (52). We focused on the AP-1 TG**A**GTCA consensus motif which contained a central A that is both critical for TF target recognition as well as overlies the expected six-nucleotide editing window for ABE8e. The AP-1 family includes dozens of conserved bZIP TFs including the FOS, JUN, and ATF protein families which can be oncogenes or tumor suppressors in different settings, as well as can be intentionally manipulated to overcome CAR-T exhaustion (53-55). Consistent with their functional role in T-cells, c-FOS and c-JUN are highly expressed (16.9 TPM and 17.5 TPM, respectively, based on ENCODE RNA-seq data) (54). We visualized the well-characterized c-FOS:c-JUN heterodimer interaction with DNA to understand the relationship of the target base, editing window, and DeepLIFT contribution scores (Fig. 5E) (53, 56). At this off-target site, the target A occurs within a GAG sequence context that is the second most favorable for ABE8e editing based on our prior HEK293T experiments (Fig. 3E) and also occurs adjacent to 12 bases of perfect gRNA seed complementarity (Fig. 5E).

We *in silico* installed an A>G mutation within this cCRE and used our ChromBPNet model to predict chromatin accessibility at the edited sequence compared to reference. This single nucleotide change is predicted to significantly decrease peak accessibility (−0.871 log2FC, −40.4-fold change in local counts, Fig. 5D, gold). Moreover, model interpretation revealed that reduced accessibility is driven by complete loss of the impact scores across the entire seven nucleotide AP-1 motif due to this single nucleotide edit. This site has the largest *in silico* predicted effect of all unique off-targets overlapping with regions of open chromatin by ATAC-seq (Fig S7D).

In contrast, we identified example control *in silico* edits which are not expected to create changes to the T-cell epigenetic landscape. For example, mutation of the nearest adenine outside of this core AP-1 motif and within the editing window results in minimal changes in local contribution scores and overall predicted accessibility (0.277 log2FC, Fig. S8A). We also investigated a second independent ABE8e-WT + CD3E gRNA off-target site occurring within a DDIT3:CEBPA JASPAR motif. *In silico* mutagenesis predicts that both As within the core TGCAAT contribute minimally to accessibility at this region (0.066 and 0.033 log2FC, Fig. S8B-D). Instead, a neighboring CTCF motif drives accessibility in this region, consistent with an evident peak in ENCODE ChIP-Seq for CTCF. Thus, this DDIT3:CEBPA edit is not expected to have substantial effects on chromatin accessibility in activated T-cells. Taken together, we show how beCasKAS can uncover base editor specific editing windows that can also be used in concert with interpretable deep learning models trained on epigenetic datasets to triage cell-type specific off-target sites of predicted regulatory importance at single base-pair resolution.

## Discussion:

Here, we describe the application of beCasKAS to quantify base editor off-target sites in cell lines and in primary human cells. Unlike other methods that can detect off-targets *in cellulo*, beCasKAS directly quantifies both unwound and edited DNA. Our method only employs commercially available reagents and can be completed within a single day. beCasKAS is especially sensitive for base editor off-targets because R-loop formation must precede ssDNA deaminase editing (Fig 3A, Fig. S9), but our method can in principle be used to probe DNA for any R-loop generating genome editor.

Our results reproduce existing findings for the prevailing model of SpCas9 DNA interrogation (30, 31, 57). Mechanistically, WT SpCas9 requires a series of concerted conformational changes to catalyze HNH and RuvC nuclease domain activation to create a double-stranded break (30). When gRNA mismatches are present, the Cas9 REC2/3 lobes can conformationally prevent nuclease activation (58). In contrast, engineered base editors are composed of two tethered proteins without evolved regulation of these conformational checkpoints. As a result, the widely deployed ABE8e exhibits significant potential for mutagenesis, as fully non-complementary substrates can even be unwound and deaminated *in vitro* (33). Given this knowledge, our study begs the question of whether detection of an off-target R-loop or a bona-fide off-target edit needs to be seen before a given base editor and gRNA pair are blacklisted. We anticipate the answer will depend heavily on the genomic context. Depending on the setting and base editor used, the relative prevalence of off-target R-loops to off-target edits can range from 74 to 1508-fold (Fig. 3F), although we note that editing is more sensitive to sequencing depth than R-loop formation (Fig. S9). We provide two examples of R-loop formation without clear off-target editing that warrant further investigation: cCREs containing an AP-1 family motif or a Ddit3-CEBPa motif (Fig. 5B-E, Fig. S8).

To explore difficult-to-annotate intergenic off-target sites, we used a cell-type specific deep learning approach to predict the effect of the exact A>G mutations in question on the epigenome. Our results highlight the value of interpretable deep learning strategies for triaging potential off-targets, as our base-resolution model can be easily used to test unintended edits found in human T-cells by any other off-target identification methodology. As models improve and epigenetic measurements grow in size, we anticipate the use of similar approaches to model consequences of non-coding off-target edits on downstream steps of gene regulation including expression. The accurate annotation of non-coding edits will be critical for selecting potent and specific therapeutic gene editors, as non-coding off-target edits could have effect sizes as large as the intentional on-target editing of non-coding regions, such as the BCL11A enhancer (Figure S6D) (51).

Our exploration of base editor therapeutic dose is important for three reasons. First, in comparing the mRNA primary T-cell data to the plasmid HEK293T data, we show that the same ABE8e editor + gRNA can result in orders of magnitude change in off-target site generation (115,355 / 339 ~ 340-fold, Fig. 3B vs 4C), underscoring the importance of FDA recommendations to deploy *in cellulo* assays that accurately recapitulate therapeutic settings. Second, we show that a significant proportion of both editing and unwinding at low-affinity off-target sites can be quantitatively eliminated through careful editor dose choice (Fig. 4C). Finally, dosing a therapeutic candidate at levels exceeding the therapeutic dose might provide a more conservative estimate of an off-target “worse case scenario” for a given model system.

Finally, we note that we deployed beCasKAS to quantify gRNA-dependent off-target sites, rather than potential gRNA-independent off-target sites that may occur due to deaminase activity on ssDNA that exists natively in the nucleus as described by prior studies (15, 26). However, our approach for optimizing primary-cell beCasKAS (Fig. S2) by pharmacologically inducing G1-arrest in activated T-cells might also minimize gRNA-independent off-target editing by limiting ssDNA in replication forks (59). We speculate that other pharmacological perturbations might further limit gRNA-independent off-target editing and are actively investigating alternative gene editing workflows with these safeguards in mind.

## Supplementary Material

1

Materials and Methods

Figs. S1 to S9

Tables S1 to S3

## References

[1] DeFrancesco L. Precision gene editing medicine makes history, and it’s just getting started. Nat. Biotechnol. 43, 1019–1022 (2025). 40588552 10.1038/s41587-025-02741-6

[2] Center for Biologics Evaluation and Research, Food and Drug Adminstration, Human Gene Therapy Products Incorporating Human Genome Editing Guidance for Industry (2024). https://www.fda.gov/regulatory-information/search-fda-guidance-documents/human-gene-therapy-products-incorporating-human-genome-editing.

[3] Bae S., Park J., Kim J.-S., Cas-OFFinder: a fast and versatile algorithm that searches for potential off-target sites of Cas9 RNA-guided endonucleases. Bioinformatics 30, 1473–1475 (2014). 24463181 10.1093/bioinformatics/btu048 PMC4016707

[4] Petri K., Kim D. Y., Sasaki K. E., Canver M. C., Wang X., Shah H., Lee H., Horng J. E., Clement K., Iyer S., Garcia S. P., Guo J. A., Newby G. A., Pinello L., Liu D. R., Aryee M. J., Musunuru K., Keith Joung J., Pattanayak V., Global-scale CRISPR gene editor specificity profiling by ONE-seq identifies population-specific, variant off-target effects, bioRxiv (2021). 10.1101/2021.04.05.438458.

[5] Lazzarotto C. R., Katta V., Li Y., Urbina E., Lee G., Tsai S. Q., CHANGE-seq-BE enables simultaneously sensitive and unbiased in vitro profiling of base editor genome-wide activity, bioRxivorg (2024). 10.1101/2024.03.28.586621.

[6] Liang P., Xie X., Zhi S., Sun H., Zhang X., Chen Y., Chen Y., Xiong Y., Ma W., Liu D., Huang J., Songyang Z., Genome-wide profiling of adenine base editor specificity by EndoV-seq. Nat. Commun. 10, 67 (2019). 30622278 10.1038/s41467-018-07988-z PMC6325126

[7] Kim D., Bae S., Park J., Kim E., Kim S., Yu H. R., Hwang J., Kim J.-I., Kim J.-S., Digenome-seq: genome-wide profiling of CRISPR-Cas9 off-target effects in human cells. Nat. Methods 12, 237–43, 1 p following 243 (2015). 25664545 10.1038/nmeth.3284

[8] Cameron P., Fuller C. K., Donohoue P. D., Jones B. N., Thompson M. S., Carter M. M., Gradia S., Vidal B., Garner E., Slorach E. M., Lau E., Banh L. M., Lied A. M., Edwards L. S., Settle A. H., Capurso D., Llaca V., Deschamps S., Cigan M., Young J. K., May A. P., Mapping the genomic landscape of CRISPR-Cas9 cleavage. Nat. Methods 14, 600–606 (2017). 28459459 10.1038/nmeth.4284

[9] Zhu M., Xu R., Yuan J., Wang J., Ren X., Cong T., You Y., Ju A., Xu L., Wang H., Zheng P., Tao H., Lin C., Yu H., Du J., Lin X., Xie W., Li Y., Lan X., Tracking-seq reveals the heterogeneity of off-target effects in CRISPR-Cas9-mediated genome editing. Nat. Biotechnol. 43, 799–810 (2025). 38956324 10.1038/s41587-024-02307-y

[10] Zou R. S., Liu Y., Gaido O. E. R., Konig M. F., Mog B. J., Shen L. L., Aviles-Vazquez F., Marin-Gonzalez A., Ha T., Improving the sensitivity of in vivo CRISPR off-target detection with DISCOVER-Seq. Nat. Methods 20, 706–713 (2023). 37024653 10.1038/s41592-023-01840-z PMC10172116

[11] Tsai S. Q., Zheng Z., Nguyen N. T., Liebers M., Topkar V. V., Thapar V., Wyvekens N., Khayter C., Iafrate A. J., Le L. P., Aryee M. J., Joung J. K., GUIDE-seq enables genome-wide profiling of off-target cleavage by CRISPR-Cas nucleases. Nat. Biotechnol. 33, 187–197 (2015). 25513782 10.1038/nbt.3117 PMC4320685

[12] Yan W. X., Mirzazadeh R., Garnerone S., Scott D., Schneider M. W., Kallas T., Custodio J., Wernersson E., Li Y., Gao L., Federova Y., Zetsche B., Zhang F., Bienko M., Crosetto N., BLISS is a versatile and quantitative method for genome-wide profiling of DNA double-strand breaks. Nat. Commun. 8, 15058 (2017). 28497783 10.1038/ncomms15058 PMC5437291

[13] Lei Z., Meng H., Lv Z., Liu M., Zhao H., Wu H., Zhang X., Liu L., Zhuang Y., Yin K., Yan Y., Yi C., Detect-seq reveals out-of-protospacer editing and target-strand editing by cytosine base editors. Nat. Methods 18, 643–651 (2021). 34099937 10.1038/s41592-021-01172-w

[14] Yuan K., Xi X., Han S., Han J., Zhao B., Wei Q., Zhou X., Selict-seq profiles genome-wide off-target effects in adenosine base editing. Nucleic Acids Res 53 (2025). 10.1093/nar/gkaf281 PMC11983105 40207628

[15] Zuo E., Sun Y., Wei W., Yuan T., Ying W., Sun H., Yuan L., Steinmetz L. M., Li Y., Yang H., Cytosine base editor generates substantial off-target single-nucleotide variants in mouse embryos. Science 364, 289–292 (2019). 30819928 10.1126/science.aav9973 PMC7301308

[16] Jin S., Zong Y., Gao Q., Zhu Z., Wang Y., Qin P., Liang C., Wang D., Qiu J.-L., Zhang F., Gao C., Cytosine, but not adenine, base editors induce genome-wide off-target mutations in rice. Science 364, 292–295 (2019). 30819931 10.1126/science.aaw7166

[17] Marinov G. K., Kim S. H., Bagdatli S. T., Higashino S. I., Trevino A. E., Tycko J., Wu T., Bintu L., Bassik M. C., He C., Kundaje A., Greenleaf W. J., CasKAS: direct profiling of genome-wide dCas9 and Cas9 specificity using ssDNA mapping. Genome Biol. 24, 85 (2023). 37085898 10.1186/s13059-023-02930-z PMC10120127

[18] Komor A. C., Kim Y. B., Packer M. S., Zuris J. A., Liu D. R., Programmable editing of a target base in genomic DNA without double-stranded DNA cleavage. Nature 533, 420–424 (2016). 27096365 10.1038/nature17946 PMC4873371

[19] Gaudelli N. M., Komor A. C., Rees H. A., Packer M. S., Badran A. H., Bryson D. I., Liu D. R., Programmable base editing of A•T to G•C in genomic DNA without DNA cleavage. Nature 551, 464–471 (2017). 29160308 10.1038/nature24644 PMC5726555

[20] Gong S., Yu H. H., Johnson K. A., Taylor D. W., DNA Unwinding Is the Primary Determinant of CRISPR-Cas9 Activity. Cell Rep 22, 359–371 (2018). 29320733 10.1016/j.celrep.2017.12.041 PMC11151164

[21] Wu X., Scott D. A., Kriz A. J., Chiu A. C., Hsu P. D., Dadon D. B., Cheng A. W., Trevino A. E., Konermann S., Chen S., Jaenisch R., Zhang F., Sharp P. A., Genome-wide binding of the CRISPR endonuclease Cas9 in mammalian cells. Nat. Biotechnol. 32, 670–676 (2014). 24752079 10.1038/nbt.2889 PMC4145672

[22] Kuscu C., Arslan S., Singh R., Thorpe J., Adli M., Genome-wide analysis reveals characteristics of off-target sites bound by the Cas9 endonuclease. Nat. Biotechnol. 32, 677–683 (2014). 24837660 10.1038/nbt.2916

[23] Wu T., Lyu R., You Q., He C., Kethoxal-assisted single-stranded DNA sequencing captures global transcription dynamics and enhancer activity in situ. Nat. Methods 17, 515–523 (2020). 32251394 10.1038/s41592-020-0797-9 PMC7205578

[24] Wang X., Li J., Wang Y., Yang B., Wei J., Wu J., Wang R., Huang X., Chen J., Yang L., Efficient base editing in methylated regions with a human APOBEC3A-Cas9 fusion. Nat. Biotechnol. 36, 946–949 (2018). 30125268 10.1038/nbt.4198

[25] Richter M. F., Zhao K. T., Eton E., Lapinaite A., Newby G. A., Thuronyi B. W., Wilson C., Koblan L. W., Zeng J., Bauer D. E., Doudna J. A., Liu D. R., Phage-assisted evolution of an adenine base editor with improved Cas domain compatibility and activity. Nat. Biotechnol. 38, 883–891 (2020). 32433547 10.1038/s41587-020-0453-z PMC7357821

[26] Buisson R., Langenbucher A., Bowen D., Kwan E. E., Benes C. H., Zou L., Lawrence M. S., Passenger hotspot mutations in cancer driven by APOBEC3A and mesoscale genomic features. Science 364 (2019). 10.1126/science.aaw2872 PMC6731024 31249028

[27] Doman J. L., Raguram A., Newby G. A., Liu D. R., Evaluation and minimization of Cas9-independent off-target DNA editing by cytosine base editors. Nature Biotechnology 38, 620–628 (2020). 10.1038/s41587-020-0414-6 PMC7335424 32042165

[28] Zhang Y., Liu T., Meyer C. A., Eeckhoute J., Johnson D. S., Bernstein B. E., Nusbaum C., Myers R. M., Brown M., Li W., Liu X. S., Model-based analysis of ChIP-Seq (MACS). Genome Biol. 9, R137 (2008). 18798982 10.1186/gb-2008-9-9-r137 PMC2592715

[29] Altschul S. F., Gish W., Miller W., Myers E. W., Lipman D. J., Basic local alignment search tool. J. Mol. Biol. 215, 403–410 (1990). 2231712 10.1016/S0022-2836(05)80360-2

[30] Sternberg S. H., LaFrance B., Kaplan M., Doudna J. A., Conformational control of DNA target cleavage by CRISPR-Cas9. Nature 527, 110–113 (2015). 26524520 10.1038/nature15544 PMC4859810

[31] Boyle E. A., Andreasson J. O. L., Chircus L. M., Sternberg S. H., Wu M. J., Guegler C. K., Doudna J. A., Greenleaf W. J., High-throughput biochemical profiling reveals sequence determinants of dCas9 off-target binding and unbinding. Proc. Natl. Acad. Sci. U. S. A. 114, 5461–5466 (2017). 28495970 10.1073/pnas.1700557114 PMC5448226

[32] Berríos K. N., Barka A., Gill J., Serrano J. C., Bailer P. F., Parker J. B., Evitt N. H., Gajula K. S., Shi J., Kohli R. M., Cooperativity between Cas9 and hyperactive AID establishes broad and diversifying mutational footprints in base editors. Nucleic Acids Res. 52, 2078–2090 (2024). 38261989 10.1093/nar/gkae024 PMC10899762

[33] Lapinaite A., Knott G. J., Palumbo C. M., Lin-Shiao E., Richter M. F., Zhao K. T., Beal P. A., Liu D. R., Doudna J. A., DNA capture by a CRISPR-Cas9-guided adenine base editor. Science 369, 566–571 (2020). 32732424 10.1126/science.abb1390 PMC8598131

[34] Love M. I., Huber W., Anders S., Moderated estimation of fold change and dispersion for RNA-seq data with DESeq2. Genome Biol. 15, 550 (2014). 25516281 10.1186/s13059-014-0550-8 PMC4302049

[35] Heinz S., Benner C., Spann N., Bertolino E., Lin Y. C., Laslo P., Cheng J. X., Murre C., Singh H., Glass C. K., Simple combinations of lineage-determining transcription factors prime cis-regulatory elements required for macrophage and B cell identities. Mol. Cell 38, 576–589 (2010). 20513432 10.1016/j.molcel.2010.05.004 PMC2898526

[36] Kohli R. M., Abrams S. R., Gajula K. S., Maul R. W., Gearhart P. J., Stivers J. T., A portable hot spot recognition loop transfers sequence preferences from APOBEC family members to activation-induced cytidine deaminase. J Biol Chem 284, 22898–22904 (2009). 19561087 10.1074/jbc.M109.025536 PMC2755697

[37] Wu Y., Xiao Y.-L., Tang W., High-precision cytosine base editors by evolving nucleic-acid-recognition hotspots in deaminase. Nature Biotechnology, 1–15 (2025). 10.1038/s41587-025-02678-w PMC12240478 40624252

[38] Schutsky E. K., Nabel C. S., Davis A. K. F., DeNizio J. E., Kohli R. M., APOBEC3A efficiently deaminates methylated, but not TET-oxidized, cytosine bases in DNA. Nucleic Acids Res. 45, 7655–7665 (2017). 28472485 10.1093/nar/gkx345 PMC5570014

[39] Chiesa R., Georgiadis C., Syed F., Zhan H., Etuk A., Gkazi S. A., Preece R., Ottaviano G., Braybrook T., Chu J., Kubat A., Adams S., Thomas R., Gilmour K., O’Connor D., Vora A., Qasim W., Base-Edited CAR T Group, Base-edited CAR7 T cells for relapsed T-cell acute lymphoblastic leukemia. N. Engl. J. Med. 389, 899–910 (2023). 37314354 10.1056/NEJMoa2300709

[40] Engel N. W., Steinfeld I., Ryan D., Anupindi K., Kim S., Wellhausen N., Chen L., Wilkins K., Baker D. J., Rommel P. C., Jarocha D., Gohil M., Zhang Q., Milone M. C., Fraietta J. A., Davis M., Young R. M., June C. H., Quadruple adenine base-edited allogeneic CAR T cells outperform CRISPR/Cas9 nuclease-engineered T cells. Proc. Natl. Acad. Sci. U. S. A. 122, e2427216122 (2025). 40324075 10.1073/pnas.2427216122 PMC12107175

[41] Gaudelli N. M., Lam D. K., Rees H. A., Solá-Esteves N. M., Barrera L. A., Born D. A., Edwards A., Gehrke J. M., Lee S.-J., Liquori A. J., Murray R., Packer M. S., Rinaldi C., Slaymaker I. M., Yen J., Young L. E., Ciaramella G., Directed evolution of adenine base editors with increased activity and therapeutic application. Nat. Biotechnol. 38, 892–900 (2020). 32284586 10.1038/s41587-020-0491-6

[42] Walton R. T., Christie K. A., Whittaker M. N., Kleinstiver B. P., Unconstrained genome targeting with near-PAMless engineered CRISPR-Cas9 variants. Science 368, 290–296 (2020). 32217751 10.1126/science.aba8853 PMC7297043

[43] Shi H., Al-Sayyad N., Wasko K. M., Trinidad M. I., Doherty E. E., Vohra K., Boger R. S., Colognori D., Cofsky J. C., Skopintsev P., Bryant Z., Doudna J. A., Rapid two-step target capture ensures efficient CRISPR-Cas9-guided genome editing. Mol. Cell 85, 1730–1742.e9 (2025). 40273916 10.1016/j.molcel.2025.03.024 PMC12258621

[44] Hibshman G. N., Bravo J. P. K., Hooper M. M., Dangerfield T. L., Zhang H., Finkelstein I. J., Johnson K. A., Taylor D. W., Unraveling the mechanisms of PAMless DNA interrogation by SpRY-Cas9. Nat. Commun. 15, 3663 (2024). 38688943 10.1038/s41467-024-47830-3 PMC11061278

[45] Pampari A., Shcherbina A., Kvon E. Z., Kosicki M., Nair S., Kundu S., Kathiria A. S., Risca V. I., Kuningas K., Alasoo K., Greenleaf W. J., Pennacchio L. A., Kundaje A., ChromBPNet: bias factorized, base-resolution deep learning models of chromatin accessibility reveal cis-regulatory sequence syntax, transcription factor footprints and regulatory variants, bioRxiv (2025). 10.1101/2024.12.25.630221.

[46] Shrikumar A., Greenside P., Kundaje A., Learning Important Features Through Propagating Activation Differences (2017). http://arxiv.org/abs/1704.02685.

[47] Martyn G. E., Montgomery M. T., Jones H., Guo K., Doughty B. R., Linder J., Bisht D., Xia F., Cai X. S., Chen Z., Cochran K., Lawrence K. A., Munson G., Pampari A., Fulco C. P., Sahni N., Kelley D. R., Lander E. S., Kundaje A., Engreitz J. M., Rewriting regulatory DNA to dissect and reprogram gene expression. Cell 188, 3349–3366.e23 (2025). 40245860 10.1016/j.cell.2025.03.034 PMC12167154

[48] Liu B. B., Jessa S., Kim S. H., Ng Y. T., Higashino S. I., Marinov G. K., Chen D. C., Parks B. E., Li L., Nguyen T. C., Wang A. T., Wang S. K., Tan S. Y., Kosicki M., Pennacchio L. A., Ben-David E., Pasca A. M., Kundaje A., Farh K. K. H., Greenleaf W. J., Dissecting regulatory syntax in human development with scalable multiomics and deep learning, bioRxiv (2025). 10.1101/2025.04.30.651381. PMC13216069 41951735

[49] Bauer D. E., Kamran S. C., Lessard S., Xu J., Fujiwara Y., Lin C., Shao Z., Canver M. C., Smith E. C., Pinello L., Sabo P. J., Vierstra J., Voit R. A., Yuan G.-C., Porteus M. H., Stamatoyannopoulos J. A., Lettre G., Orkin S. H., An erythroid enhancer of BCL11A subject to genetic variation determines fetal hemoglobin level. Science 342, 253–257 (2013). 24115442 10.1126/science.1242088 PMC4018826

[50] Frangoul H., Locatelli F., Sharma A., Bhatia M., Mapara M., Molinari L., Wall D., Liem R. I., Telfer P., Shah A. J., Cavazzana M., Corbacioglu S., Rondelli D., Meisel R., Dedeken L., Lobitz S., de Montalembert M., Steinberg M. H., Walters M. C., Eckrich M. J., Imren S., Bower L., Simard C., Zhou W., Xuan F., Morrow P. K., Hobbs W. E., Grupp S. A., CLIMB SCD-121 Study Group, Exagamglogene Autotemcel for Severe Sickle Cell Disease. N Engl J Med 390, 1649–1662 (2024). 38661449 10.1056/NEJMoa2309676

[51] Liao J., Chen S., Hsiao S., Jiang Y., Yang Y., Zhang Y., Wang X., Lai Y., Bauer D. E., Wu Y., Therapeutic adenine base editing of human hematopoietic stem cells. Nat Commun 14, 207 (2023). 36639729 10.1038/s41467-022-35508-7 PMC9839747

[52] Rauluseviciute I., Riudavets-Puig R., Blanc-Mathieu R., Castro-Mondragon J. A., Ferenc K., Kumar V., Lemma R. B., Lucas J., Chèneby J., Baranasic D., Khan A., Fornes O., Gundersen S., Johansen M., Hovig E., Lenhard B., Sandelin A., Wasserman W. W., Parcy F., Mathelier A., JASPAR 2024: 20th anniversary of the open-access database of transcription factor binding profiles. Nucleic Acids Res 52, D174–D182 (2024). 37962376 10.1093/nar/gkad1059 PMC10767809

[53] Shaulian E., Karin M., AP-1 in cell proliferation and survival. Oncogene 20, 2390–2400 (2001). 11402335 10.1038/sj.onc.1204383

[54] ENCODE Project Consortium, An integrated encyclopedia of DNA elements in the human genome. Nature 489, 57–74 (2012). 22955616 10.1038/nature11247 PMC3439153

[55] Lynn R. C., Weber E. W., Sotillo E., Gennert D., Xu P., Good Z., Anbunathan H., Lattin J., Jones R., Tieu V., Nagaraja S., Granja J., de Bourcy C. F. A., Majzner R., Satpathy A. T., Quake S. R., Monje M., Chang H. Y., Mackall C. L., c-Jun overexpression in CAR T cells induces exhaustion resistance. Nature 576, 293–300 (2019). 31802004 10.1038/s41586-019-1805-z PMC6944329

[56] Glover J. N. M., Harrison S. C., Crystal structure of the heterodimeric bZIP transcription factor c-Fos–c-Jun bound to DNA. Nature 373, 257–261 (1995). 7816143 10.1038/373257a0

[57] Jiang F., Taylor D. W., Chen J. S., Kornfeld J. E., Zhou K., Thompson A. J., Nogales E., Doudna J. A., Structures of a CRISPR-Cas9 R-loop complex primed for DNA cleavage. Science 351, 867–871 (2016). 26841432 10.1126/science.aad8282 PMC5111852

[58] Pacesa M., Lin C.-H., Cléry A., Saha A., Arantes P. R., Bargsten K., Irby M. J., Allain F. H.-T., Palermo G., Cameron P., Donohoue P. D., Jinek M., Structural basis for Cas9 off-target activity. Cell 185, 4067–4081.e21 (2022). 36306733 10.1016/j.cell.2022.09.026 PMC10103147

[59] Hoopes J. I., Cortez L. M., Mertz T. M., Malc E. P., Mieczkowski P. A., Roberts S. A., APOBEC3A and APOBEC3B preferentially deaminate the lagging strand template during DNA replication. Cell Rep. 14, 1273–1282 (2016). 26832400 10.1016/j.celrep.2016.01.021 PMC4758883

[60] Pack L. R., Daigh L. H., Chung M., Meyer T., Clinical CDK4/6 inhibitors induce selective and immediate dissociation of p21 from cyclin D-CDK4 to inhibit CDK2. Nature Communications 12, 1–12 (2021). 10.1038/s41467-021-23612-z PMC8184839 34099663

[61] Clement K., Rees H., Canver M. C., Gehrke J. M., Farouni R., Hsu J. Y., Cole M. A., Liu D. R., Joung J. K., Bauer D. E., Pinello L., CRISPResso2 provides accurate and rapid genome editing sequence analysis. Nat Biotechnol 37, 224–226 (2019). 30809026 10.1038/s41587-019-0032-3 PMC6533916

[62] Lyu R., Wu T., Park G., He Y.-Y., Chen M., He C., KAS-Analyzer: a novel computational framework for exploring KAS-seq data. Bioinform. Adv. 3, vbad121 (2023). 37745002 10.1093/bioadv/vbad121 PMC10516523

[63] Langmead B., Salzberg S. L., Fast gapped-read alignment with Bowtie 2. Nat. Methods 9, 357–359 (2012). 22388286 10.1038/nmeth.1923 PMC3322381

[64] Ramírez F., Dündar F., Diehl S., Grüning B. A., Manke T., deepTools: a flexible platform for exploring deep-sequencing data. Nucleic Acids Res. 42, W187–91 (2014). 24799436 10.1093/nar/gku365 PMC4086134

[65] Sievers F., Higgins D. G., Clustal Omega for making accurate alignments of many protein sequences. Protein Sci 27, 135–145 (2018). 28884485 10.1002/pro.3290 PMC5734385

[66] Goddard T. D., Huang C. C., Meng E. C., Pettersen E. F., Couch G. S., Morris J. H., Ferrin T. E., UCSF ChimeraX: Meeting modern challenges in visualization and analysis. Protein Sci 27, 14–25 (2018). 28710774 10.1002/pro.3235 PMC5734306

[67] McKenna A., Hanna M., Banks E., Sivachenko A., Cibulskis K., Kernytsky A., Garimella K., Altshuler D., Gabriel S., Daly M., DePristo M. A., The Genome Analysis Toolkit: a MapReduce framework for analyzing next-generation DNA sequencing data. Genome Res. 20, 1297–1303 (2010). 20644199 10.1101/gr.107524.110 PMC2928508

[68] Lin Y.-C., Boone M., Meuris L., Lemmens I., Van Roy N., Soete A., Reumers J., Moisse M., Plaisance S., Drmanac R., Chen J., Speleman F., Lambrechts D., Van de Peer Y., Tavernier J., Callewaert N., Genome dynamics of the human embryonic kidney 293 lineage in response to cell biology manipulations. Nat. Commun. 5, 4767 (2014). 25182477 10.1038/ncomms5767 PMC4166678

[69] Manders F., Brandsma A. M., de Kanter J., Verheul M., Oka R., van Roosmalen M. J., van der Roest B., van Hoeck A., Cuppen E., van Boxtel R., MutationalPatterns: the one stop shop for the analysis of mutational processes. BMC Genomics 23, 134 (2022). 35168570 10.1186/s12864-022-08357-3 PMC8845394

[70] Gu Z., Gu L., Eils R., Schlesner M., Brors B., circlize Implements and enhances circular visualization in R. Bioinformatics 30, 2811–2812 (2014). 24930139 10.1093/bioinformatics/btu393

[71] Corces M. R., Buenrostro J. D., Wu B., Greenside P. G., Chan S. M., Koenig J. L., Snyder M. P., Pritchard J. K., Kundaje A., Greenleaf W. J., Majeti R., Chang H. Y., Lineage-specific and single-cell chromatin accessibility charts human hematopoiesis and leukemia evolution. Nat Genet 48, 1193–1203 (2016). 27526324 10.1038/ng.3646 PMC5042844

[72] Marinov G. K., Shipony Z., Kundaje A., Greenleaf W. J., Genome-Wide Mapping of Active Regulatory Elements Using ATAC-seq. Methods in molecular biology (Clifton, N.J.) 2611 (2023). 10.1007/978-1-0716-2899-7_1 36807060

[73] Marinov G. K., Shipony Z., Interrogating the Accessible Chromatin Landscape of Eukaryote Genomes Using ATAC-seq. Methods in molecular biology (Clifton, N.J.) 2243 (2021). 10.1007/978-1-0716-1103-6_10 33606259

[74] Langmead B., Trapnell C., Pop M., Salzberg S. L., Ultrafast and memory-efficient alignment of short DNA sequences to the human genome. Genome Biology 10, 1–10 (2009). 10.1186/gb-2009-10-3-r25 PMC2690996 19261174

[75] Schreiber J. tangermeme: A toolkit for understanding cis-regulatory logic using deep learning models, bioRxiv (2025). 10.1101/2025.08.08.669296.

