# Supplementary material for: Sensitive, direct detection of non-coding off-target base editor unwinding and editing in primary cells
Source: bioRxiv. 2025 Sep 25:2025.09.25.678665. Preprint. [Version 1] doi: 10.1101/2025.09.25.678665 (PMC12485731; doi:10.1101/2025.09.25.678665)
Supplement: 1 [file NIHPP2025.09.25.678665v1-supplement-1.pdf]

bioRxiv preprint doi: https://doi.org/10.1101/2025.09.25.678665; this version posted September 25, 2025. The copyright holder for this preprint (which was not certified by peer review) is the author/funder, who has granted bioRxiv a license to display the preprint in perpetuity. It is made available under aCC-BY 4.0 International license.

Supplementary Materials:

Materials and Methods:

Cell Culture:

HEK293T cells were cultured in DMEM + 10% FBS media. All cells were incubated at 37°C with 5% CO2. Primary human CD4+/CD25- T cells were isolated from a leukopak (27 year old female donor, non-smoker, BMI < 30, non-mobilized apheresis collection, consented for full genomic release by STEMCELL Technologies) by negative selection using the manufacturer's protocol. Cells were stored in liquid nitrogen at 18 million cells per aliquot in 1 mL of 90% FBS with 10% DMSO.

Cloning

pCMV-hA3A-eBE-Y130F was a gift from Jia Chen (Addgene 113423). Q5 Site Directed Mutagenesis (NEB) was performed as in manufacturer instructions to reintroduce the WT Y130 residue into the eBE plasmid. pCMV-ABE8e was a gift from David Liu (Addgene 138489). pCMV-SpRY-ABE8e was a gift from David Liu (Addgene # 185671). pFYF1548 EMX1 was a gift from Keith Joung (Addgene 47508). Q5 Site Directed Mutagenesis (NEB) was performed to obtain the HEK4 gRNA plasmid. For both ABE8e and ABE8e-SpRY, the coding sequence was PCR amplified from the appropriate plasmid and cloned into a linearized plasmid template with 5'-UTR, 3'-UTR, and poly(A) sequence (Takara 6143). Midipreps (Qiagen) with elution in 10 mM Tris HCl, pH 8.0 were performed to obtain plasmids used in DNA transfection and *in vitro* mRNA transcription. Plasmid integrity was verified by nanopore sequencing (Plasmidsaurus). Plasmids with a poly(A) tail were additionally Sanger sequenced to confirm presence of >100 consecutive adenines (Azenta). Primer and PCR conditions are provided in Table S1.

HEK293T Transfection

300,000 cells were seeded per well in a 12-well plate the day before transfection to a final volume of 1 mL media per well. Cells were ~80% confluent the next day. 1.2 µg of base editor plasmid, 400 ng of gRNA plasmid, and 100 ng pMax-GFP plasmid was transfected in each condition. pUC19 plasmid was used to fill total plasmid transfection DNA up to 1.6 µg in conditions without gRNA. 1.6 µg plasmid DNA was diluted in 100 µL Opti-MEM and 6.0 Fugene HD Transfection reagent at 10 min at room temperature before addition to each well. Cells were allowed to grow for 24, 48, or 72 hrs before kethoxal labelling.

mRNA generation

50 µg of midiprep plasmid DNA was digested in 250 µL reaction volume with rCutSmart Buffer and 20 units of HindIII-HF (NEB) for 1 hr at 37°C. A 0.6x left-sided SPRI bead purification

bioRxiv preprint doi: https://doi.org/10.1101/2025.09.25.678665; this version posted September 25, 2025. The copyright holder for this preprint (which was not certified by peer review) is the author/funder, who has granted bioRxiv a license to display the preprint in perpetuity. It is made available under aCC-BY 4.0 International license.

(Beckman) was performed, and DNA was eluted in 20 µL IDTE (10 mM Tris HCl, pH 8.0, 0.1 mM EDTA). DNA was quantified by Qubit 1x dsDNA assay. *In vitro* transcription (IVT) was performed with T7 PrimeCap polymerase (Takara 6144) as in manufacturer instructions with cotranscriptional capping (CleanCap AG, TriLink) and replacing uridine triphosphate with 5-methoxyuridine triphosphate (5moU, TriLink). IVT reactions were incubated at 37°C for 2 hrs with additional brief handmixing after 30 min and 1 hr. 80 U DNase I (Takara) was added and incubated at 37°C for 15 min. Spin columns were used for mRNA purification (NEB Monarch Spin RNA Cleanup Kit) and RNA was eluted in 50 µL IDTE. RNA was quantified by nanodrop and integrity verified by BioAnalyzer (Agilent RNA Nano).

Primary T-cell electroporation

T cells (~18 million/mL) were thawed in ImmunoCult basal media (STEMCELL 10981) supplemented with 20% FBS and 20 IU/mL of DNase I (Worthington LS002007), centrifuged at 300 g, and resuspended at 1 million cells/mL in a T75 uncoated flask (Corning) containing complete media comprising basal ImmunoCult media supplemented with recombinant IL-2 (50 IU/mL), Pen/Strep (100 U/mL), and CD3/CD28/CD2 activator (STEMCELL 10970). T-cell media always contained IL2 and Pen/Strep at the same concentrations in subsequent steps. On day 3 after thawing, cells were diluted in T-cell media to a concentration of 150,000 cells / mL. On day 5, 1 million cells were diluted in 20 µL of electroporation buffer (Lonza, 90% P3, 10% Supplement), 1 µL 5moU-modified base editor mRNA (1 pmol / µL in IDTE, except for dilution series), and 1 µL sgRNA (30 pmol / µL in IDTE, IDT Alt-R™, standard desalting, RUO-grade, Table S2) before electroporation with a Lonza 4D-Nucleofector and pulse code EO-115. Cells were recovered for 5 min at room temperature before addition of 100 µL T-cell media per cuvette. Cells from 4 cuvettes were pooled (4 million cells electroporated per condition) into single wells of a 12-well plate and diluted to a 1.6 mL final volume per well. After 3 hrs, cells were G1 arrested with 5 µM palbociclib (Selleck) before allowing to grow overnight for ~20 hrs before further processing (*60*).

beCasKAS experiments

beCasKAS experiments were carried out as previously described (*17*) with some modifications. 1 µL of 500 mM N_3-kethoxal (APE-Bio, dissolved in 100% DMSO) was diluted to 5 mM in 100 µL media per sample and pre-heated to 37°C. For HEK293T cells, media was removed from cells and a PBS wash was performed. Cells were trypsinized, and trypsin was quenched with media before transfer to 1.7-mL microfuge tubes. Cells were pelleted for 5 min at 500 g and resuspended in 100 µL of 5 mM N_3-kethoxal, and cells were labeled at 37°C in a Thermomixer at 500 rpm for 10 min.

For primary T cells, 20 U DNase I (NEB) and 0.8 U Proteinase K (NEB) were added to the media before harvesting, and cells were placed back in the incubator for 30 min at 37°C. Cells were transferred to 1.7 mL microfuge tubes. Cells were pelleted for 5 min at 500 g and washed once with PBS. Cells were resuspended in Stain Buffer with FBS (BD Biosciences) and DAPI (final

bioRxiv preprint doi: https://doi.org/10.1101/2025.09.25.678665; this version posted September 25, 2025. The copyright holder for this preprint (which was not certified by peer review) is the author/funder, who has granted bioRxiv a license to display the preprint in perpetuity. It is made available under aCC-BY 4.0 International license.

concentration: 1 ug/mL). Cells were strained with a 35 µm mesh cap and collected in polystyrene test tubes (Fisher) before flow sorting for DAPI-negative single cells (FACSAria Fusion, Fig. S3). Cells were pelleted for 5 min at 500 g and the supernatant was removed. The cells were then resuspended in 100 µL of 5 mM N_3-kethoxal and labeled at 37°C and 500 rpm for 10 min in a Thermomixer.

Genomic DNA was immediately obtained using spin column purification (NEB Monarch) and eluted in 100 µL 25 mM Sodium Borate, pH 7 (Teknova) with 10 mM EDTA. gDNA was quantified by Qubit 1x dsDNA reagent. Click reaction was carried out using 87.5 µL of purified gDNA (5-15 ng/µL), 2.5 µL 20 mM DBCO-PEG4 (Sigma) and 10 µL 10x PBS, incubated at 37°C and 500 rpm for 90 min in a Thermomixer. DNA was then purified again using a spin column (NEB Monarch) and eluted in 130 µL 25 mM Sodium Borate, pH 7 without EDTA. DNA was then sheared down to 150-400 bp using a Covaris E220 (peak incident power 175 W, duty factor 10%, cycles per burst 200, treatment time 200 seconds). For biotin pull down, 10 µL of Dynabeads MyOne Streptavidin C1 beads (Thermo) were used per sample. Beads were first washed with 1x B&W before resuspension in 120 µL 2x B&W (10 mM Tris HCl pH 7.5, 1 mM EDTA, 2 M NaCl, 0.1% Tween-20). A portion (10 µL) of sheared samples was saved as input. The remaining 120 µL of sheared DNA were added to the resuspended beads and incubated for 15 min with gentle rotation at room temperature. The samples were placed on a magnet and the supernatant was removed. DNA was washed a total of four times with 1x B&W. During each wash, the sample was incubated in a Thermomixer at 55°C and 1000 rpm for 1 min before placing it on a magnet rack and removing the supernatant. DNA was eluted by adding 16 µL nuclease-free H_2O to each sample (and 6 µL H_2O to input samples) and heated to 95°C for 10 min. 15 µL used as input to library preparation. Libraries were prepared using the IDT xGen™ Methyl-Seq Lib Prep kit (10009860) following all steps for <10 ng input gDNA manufacturer instructions (17 PCR cycles). Libraries were quantified using the Qubit 1x dsDNA kit and fragment distribution visualized by Tapestation before pooling to 4 nM in IDTE buffer (Fig. S2E).

Libraries were sequenced using a NextSeq 550 High Output kit (in 2 x 150 bp format) following manufacturer instructions or through Novogene using one lane of a NovaSeq X 10B kit (2 x 150 bp) with a goal of obtaining 20-40 million reads per library. We note that 2x150-bp sequencing is often necessary to capture the middle of the DNA fragment containing the Cas9 binding site, although more stringent shearing and size selection could be used if shorter sequencing reads are desired.

Cell cycle analysis

T-cells were thawed and activated as described above. On day 3 after activation, cells were diluted to 150,000 cells / mL and 500 µL was plated in individual wells of a 24-well plate. On day 4, palbociclib or equivalent concentration of DMSO was added to the media and cells were blocked overnight for ~20 hours. The next day, BrdU and 7-AAD based cell cycle labelling was performed

bioRxiv preprint doi: https://doi.org/10.1101/2025.09.25.678665; this version posted September 25, 2025. The copyright holder for this preprint (which was not certified by peer review) is the author/funder, who has granted bioRxiv a license to display the preprint in perpetuity. It is made available under aCC-BY 4.0 International license.

according to manufacturer instructions (BD Pharmingen 559619) with a 2-hour BrdU pulse (Fig. S2B).

Amplicon sequencing

Twelve amplicons were designed using the IDT rhAmpSeq software, and rhAmpSeq libraries were generated according to manufacturer instructions. Libraries were quantified using the Qubit 1x dsDNA kit and fragment distribution was evaluated using a Tapestation before pooling to 4 nM in IDTE buffer. Amplicons were analyzed using CRISPResso2 (*61*). The correlation of the R-loop allele fraction from beCasKAS and absolute allele fraction from each amplicon was calculated to compute a best fit line by linear regression, where the slope represents the average occupancy of the base editor at each binding site. Absolute mutation frequency (per genome) is computed by summing the allele fraction (*AF*) at *n* edited sites identified by DESeq2.

$$absolute\ mutation\ frequency = occupancy \cdot \sum_{i=1}^{n} AF_i$$

KAS-seq data processing

Sequencing reads were trimmed using Trim Galore! specifically removing 12 bases from the 5' end of R2 per IDT instructions. The KAS-Analyzer wrapper was used for subsequent standard steps including alignment to the hg38 version of the human genome using Bowtie2 (*62*, *63*). Reads were deduplicated using gencore (https://github.com/OpenGene/gencore) which allows for consensus duplicates to be obtained. Signal was normalized to Reads per million (RPM) to make samples sequenced to different read depths directly comparable.

The deeptools multiBigwigSummary and plotCorrelation functions were used to visualize global beCasKAS signals (*64*). Point-source sharp peaks were called using MACS2 against an Input control (q-value = 0.01) (*28*). BLAST peaks were called using the blastn-short algorithm and the gRNA sequence as the query (*29*). Clustal Omega was used for multiple sequence alignment to build a position weight matrix (*65*). The aligned nucleotides could be further processed with perbase (https://github.com/sstadick/perbase) to visualize strand-specific editing of each base editor. Protein structures are visualized in UCSF ChimeraX (*66*).

Differential peaks +/- gRNA are called using the DiffBind package using the DESeq2 algorithm (FDR < 0.05, FC > 1) and only the summit (middle 100 bp) was used to search for subsequent variants (*34*). *De novo* motif search was performed using HOMER on differential peaks (*35*). The GATK mutect2 function was used to call variants in "tumor-only" mode (*67*). Variants were atomized with bcftools and any variants aligning to alternate chromosomes were removed. For HEK293T cells experiments, previously identified somatic variants from the HEK293T cell line were removed (*68*). Variants were visualized using the MutationalPatterns R package (*69*).

bioRxiv preprint doi: https://doi.org/10.1101/2025.09.25.678665; this version posted September 25, 2025. The copyright holder for this preprint (which was not certified by peer review) is the author/funder, who has granted bioRxiv a license to display the preprint in perpetuity. It is made available under aCC-BY 4.0 International license.

For the primary T-cell mRNA dilution series, the deeptools computeMatrix and plotHeatmap functions were used to visualize gRNA-dependent R-loops generated by the highest concentration of editor (9 pmol / million). Circos plots were generated using the circlize R package (*70*).

Primary T-cell ATAC-seq

Cells were thawed and activated in the same way as for primary T cell beCasKAS. On day 3 after activation, cells were G1-blocked with 5 µM palbociclib (Selleck) before allowing them to grow overnight for ~20 hrs. The Fast-ATAC protocol was used as previously described (*71*). Briefly, cells (50-200,000) were harvested by centrifugation at 500 g for 5 minutes, and the supernatant was removed, then resuspended in 50 µl of transposase mixture (25 µl of 2× TD buffer, 2.5 µl of TDE1, 0.5 µl of 1% digitonin, and 22 µl of nuclease-free water), and incubated at 37°C for 30 min in a ThermoMixer with agitation at 1,000 rpm. The reaction was immediately stopped by adding 250 µl of PB Buffer, and DNA was purified using the MinElute Reaction Cleanup kit (QIAGEN), eluting in 20 µl of Elution Buffer. Final libraries were generated as previously described (*72*): 20 µl of DNA, 2.5 µl of each of the two indexed primers, 25 µl 2x NEBNext High-Fidelity PCR Master Mix, with an initial extension and fill-in for 5 minutes at 72°C; followed by initial denaturation for 30 sec at 98°C, and 10 cycles of 10 sec at 98°C, 30 sec at 63°C, and 30 sec at 72°C. Libraries were purified using the Qiagen MinElute kit, quantified using Qubit 1x dsDNA kit, evaluated on a TapeStation, and then sequenced on an Illumina NextSeq 550 as 2 × 38mers.

ATAC-seq data processing

Computational processing was carried out as previously described (*73*). Demultiplexed FASTQ files were mapped to the GRCh38 (hg38) assembly of the human genome as 2 × 36mers using Bowtie with the settings "-v 2 -k 2 -m 1 --best --strata" (*74*). Mitochondrial mapping reads were filtered out, and duplicate reads were removed using picard-tools MarkDuplicates. Reads were also mapped separately to the mitochondrial genome using the Bowtie settings "-v 2 -a --best --strata", to estimate the extent of mitochondrial contamination. Three technical replicates were pooled for subsequent ChromBPNet model training.

ChromBPNet model training and interpretation

ChromBPNet models are supervised convolutional neural networks trained to use 2,114-bp one hot-encoded DNA sequence in peaks and background regions to predict the accessibility profile (as a probability distribution) and total natural log counts (as a scalar value) in the central 1,000-bp window of input regions (*45*).

To define genomic regions for training ChromBPNet models, we followed our prior workflow. First, we defined a lenient set of accessible regions. Using our T-cell ATAC-seq data, we first derived pseudoreplicates. For each ATAC-seq fragment, starts and ends (corresponding to Tn5 insertion sites) were randomly allocated to each of two pseudoreplicate files, and pseudoreplicate

bioRxiv preprint doi: https://doi.org/10.1101/2025.09.25.678665; this version posted September 25, 2025. The copyright holder for this preprint (which was not certified by peer review) is the author/funder, who has granted bioRxiv a license to display the preprint in perpetuity. It is made available under aCC-BY 4.0 International license.

files were also concatenated into a total-pseudoreplicate file. Macs2 (v2.2.9.1) was used to call peaks on all three pseudoreplicate files with parameters: *-p 0.01 --shift -75 --extsize 150 --nomodel -B --SPMR --keep-dup all --call-summits*. Only peaks called on the total-pseudoreplicate which overlapped peaks called in both pseudoreplicates were retained. Peaks overlapping the GRCh38 ENCODE blacklist (ENCODE accession ENCFF356LFX) were excluded. Peak coordinates were adjusted to 1,000 bp centered at the Macs2 peak summit. Pseudoreplicates were only used for peak calling, and pseudobulk fragment files were used for downstream model training.

We used the ChromBPNet package (https://github.com/kundajelab/chrombpnet, commit a5c231) and followed the workflow described by Pampari et al (*45*). We used the command *chrombpnet prep nonpeaks* to define background regions which match the GC content of peak regions. For each cell type, we used a five-fold cross-validation scheme, where each fold (designated 0 to 4) comprised a different set of training, validation, and test chromosomes, with each chromosome in the test set of at least one fold. We used the default human chromosome folds provided with ChromBPNet (https://doi.org/10.5281/ZENODO.7445373).

ChromBPNet models use a pre-trained bias model and explain the residual accessibility not captured by Tn5 enzyme bias. We trained a bias model to learn the enzymatic bias in our ATAC-seq setting using the fold 0 chromosome split, with bias threshold factor -b 0.9 using the chrombpnet bias pipeline, which also performs model interpretation using DeepLIFT (*45*, *46*). We confirmed that the bias model learned the Tn5 motifs but not transcription factor motifs, and used this bias model to subsequently train ChromBPNet models using the chrombpnet pipeline command with the GRCh38 reference genome from ENCODE. Models were evaluated based on the Pearson and Spearman correlations between predicted and observed log counts in peaks and the Jensen-Shannon Distance between predicted and observed profiles in peaks, for peaks on held-out test-set chromosomes (Fig. S7A-B, Table S3). To generate the average predicted accessibility tracks across folds for peak regions (representing counts per base), for each region, the mean predicted profile logits across folds were softmaxed to convert them to probabilities, then scaled by the exponentiated mean predicted log counts across folds.

We performed model interpretation to determine the extent to which each nucleotide was predictive for accessibility. We ran the chrombpnet interpret command which uses the DeepLIFT algorithm to compute contribution scores for each nucleotide in the 2,114 bp input windows with respect to the predicted counts. Contribution scores were derived for each model fold for all peak regions, and the mean computed across folds. The averaged predicted accessibility profiles and contribution scores were converted to bigWig files for visualization in genome browsers, as well as used for all analyses and figures.

Prediction of variant effect using ChromBPNet models

We predicted and interpreted effects of specific noncoding variants on chromatin accessibility using trained ChromBPNet models, as we have done previously (*48*). We used the tangermeme

bioRxiv preprint doi: https://doi.org/10.1101/2025.09.25.678665; this version posted September 25, 2025. The copyright holder for this preprint (which was not certified by peer review) is the author/funder, who has granted bioRxiv a license to display the preprint in perpetuity. It is made available under aCC-BY 4.0 International license.

package (v0.4.3) for predictions and model interpretation (*75*). For each variant, we used the 1,000 bp model training peaks, and extracted the reference genome sequence for the peak which the variant overlapped. This sequence (extended equally on either side to 2,114 bp) was fed to all five fold trained T-cell ChromBPNet models, to obtain predicted accessibility profile and aggregate log counts in the peak. For each fold, to transform predicted profile logits into accessibility profiles, the profile logits were softmaxed and scaled by the exponentiated predicted log counts; and model interpretation with respect to the counts output was performed using DeepLIFT. Next, the effect allele was substituted into the sequence at the variant position, and predictions and contribution scores were obtained as for the reference sequence. For each model, we computed the variant effect as the sum of differences in per-base predicted read counts in the 100 bp window centered at variant, and computed the mean effect score across folds. We also computed the log2 fold change between predicted counts for the effect versus the non effect allele for the peak region, where a log2 fold change > 0 indicates the effect allele was predicted to increase accessibility. In figures, the mean predicted profiles and contribution scores across folds are shown.

bioRxiv preprint doi: https://doi.org/10.1101/2025.09.25.678665; this version posted September 25, 2025. The copyright holder for this preprint (which was not certified by peer review) is the author/funder, who has granted bioRxiv a license to display the preprint in perpetuity. It is made available under aCC-BY 4.0 International license.

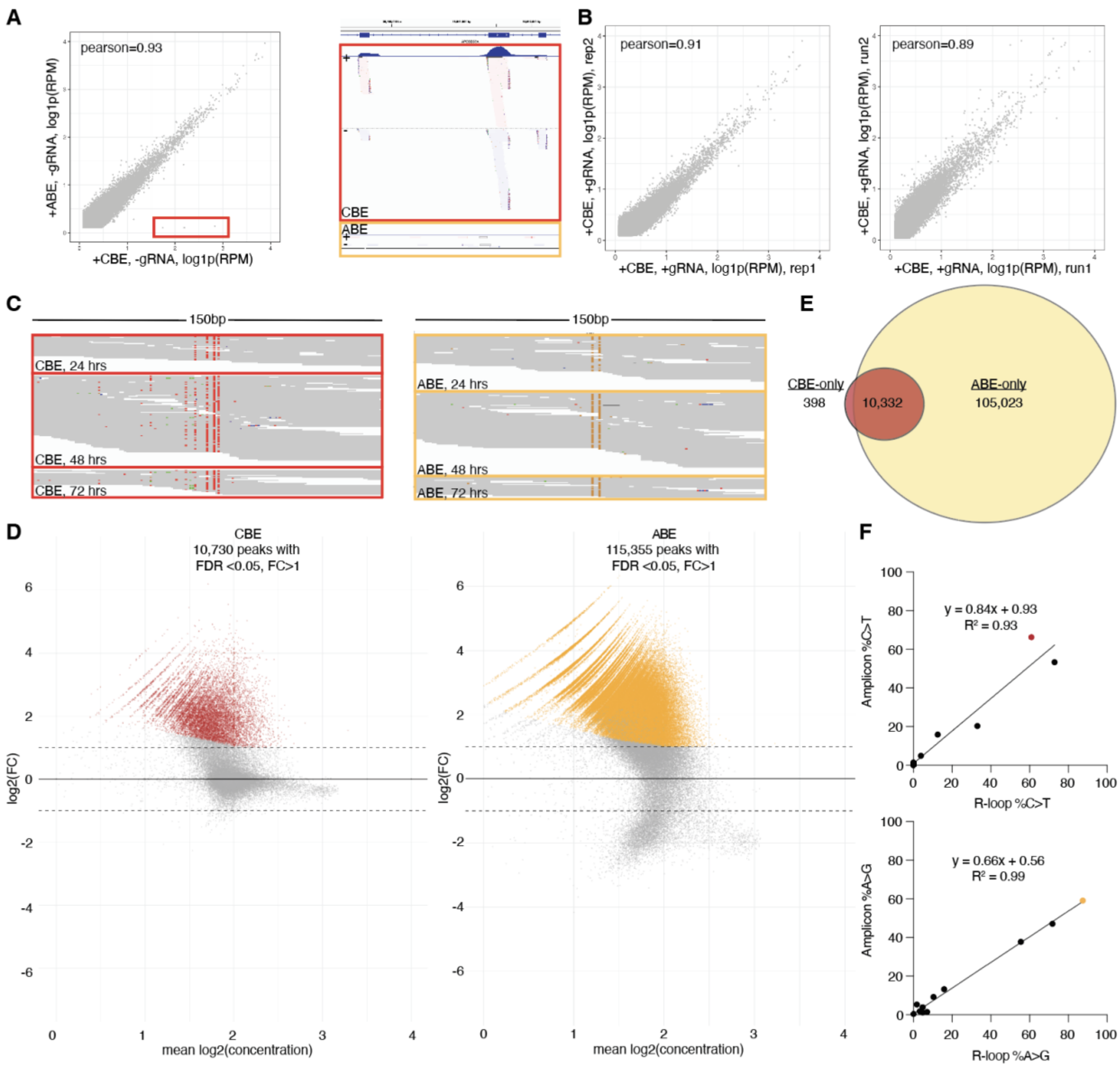

Fig. S1: HEK293T beCasKAS. A) Outlier ssDNA signal highly enriched in +CBE, -gRNA conditions and not +ABE, -gRNA conditions. IGV screenshot showing the endogenous APOBEC3A gene. **B)** Correlation of +CBE, +gRNA conditions from replicate experiments performed on the same day (left) or on independent days (right). **C)** Same representative off-target peak as in **Fig. 1** showing difference in beCasKAS reads on different days. **D)** DESeq2 generated MA-plots for discovering gRNA dependent R-loops for CBE +/- gRNA (left) and ABE +/- gRNA (right). **E)** Overlap of DESeq2 called peaks for CBE and ABE (FDR<0.05, FC>1). **F)** Correlation of allele fractions for amplicon sequencing for beCasKAS sequencing reads.

bioRxiv preprint doi: https://doi.org/10.1101/2025.09.25.678665; this version posted September 25, 2025. The copyright holder for this preprint (which was not certified by peer review) is the author/funder, who has granted bioRxiv a license to display the preprint in perpetuity. It is made available under aCC-BY 4.0 International license.

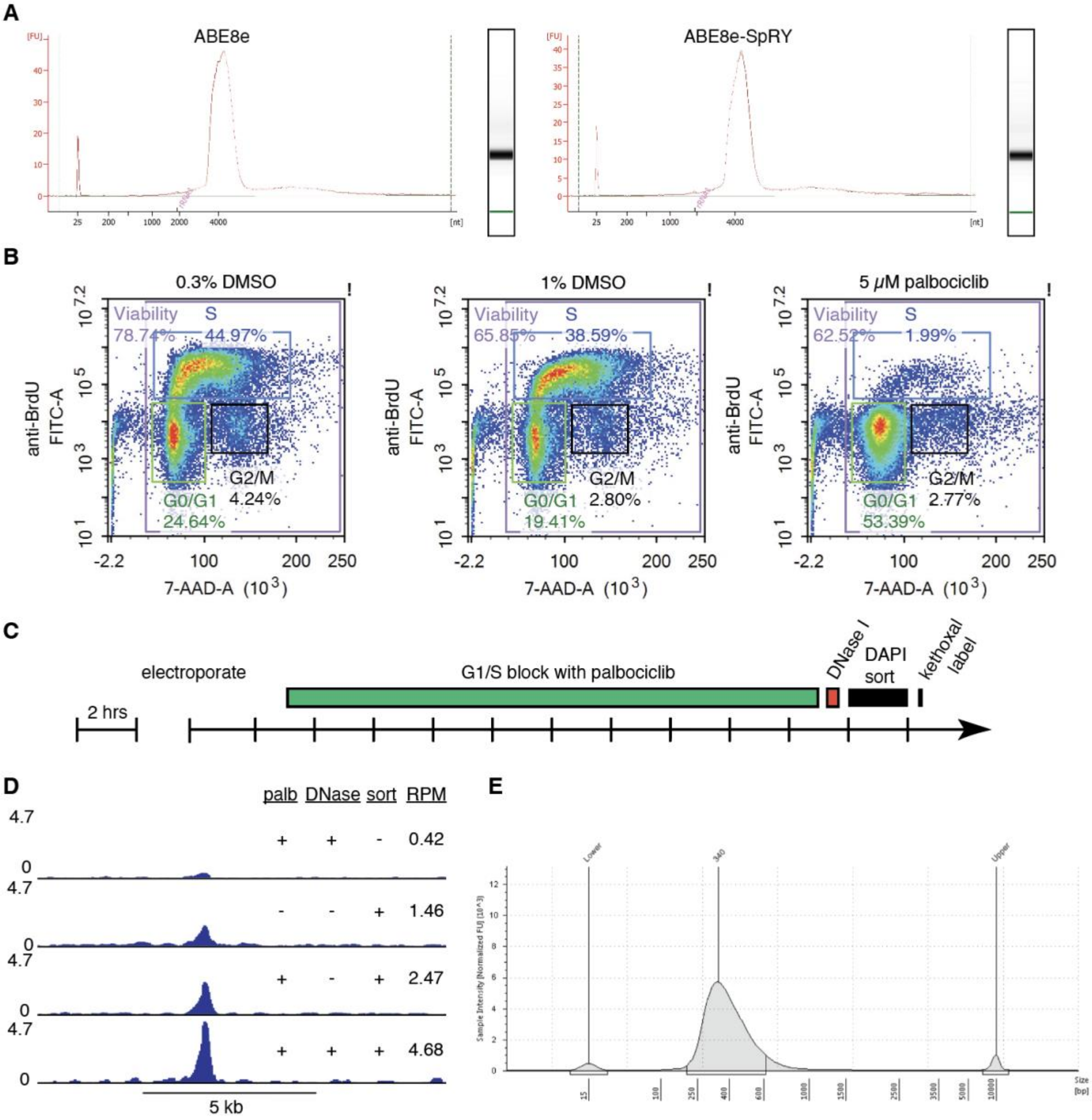

Fig. S2: Primary human T cell beCasKAS quality control and optimization. A) BioAnalyzer traces verifying purity of mRNAs used in this study. **B)** Cell cycle plots of activated T-cells treated overnight with DMSO (vehicle) or Palbociclib. **C)** Primary cell beCasKAS workflow. **D)** HEK4 on-target site against three independent variables: palbociclib G1 block , DNase I treatment, and DAPI viability sorting, which all improve ssDNA signal at on-target site. The peak RPM value is provided. **E)** Example final beCasKAS library.

bioRxiv preprint doi: https://doi.org/10.1101/2025.09.25.678665; this version posted September 25, 2025. The copyright holder for this preprint (which was not certified by peer review) is the author/funder, who has granted bioRxiv a license to display the preprint in perpetuity. It is made available under aCC-BY 4.0 International license.

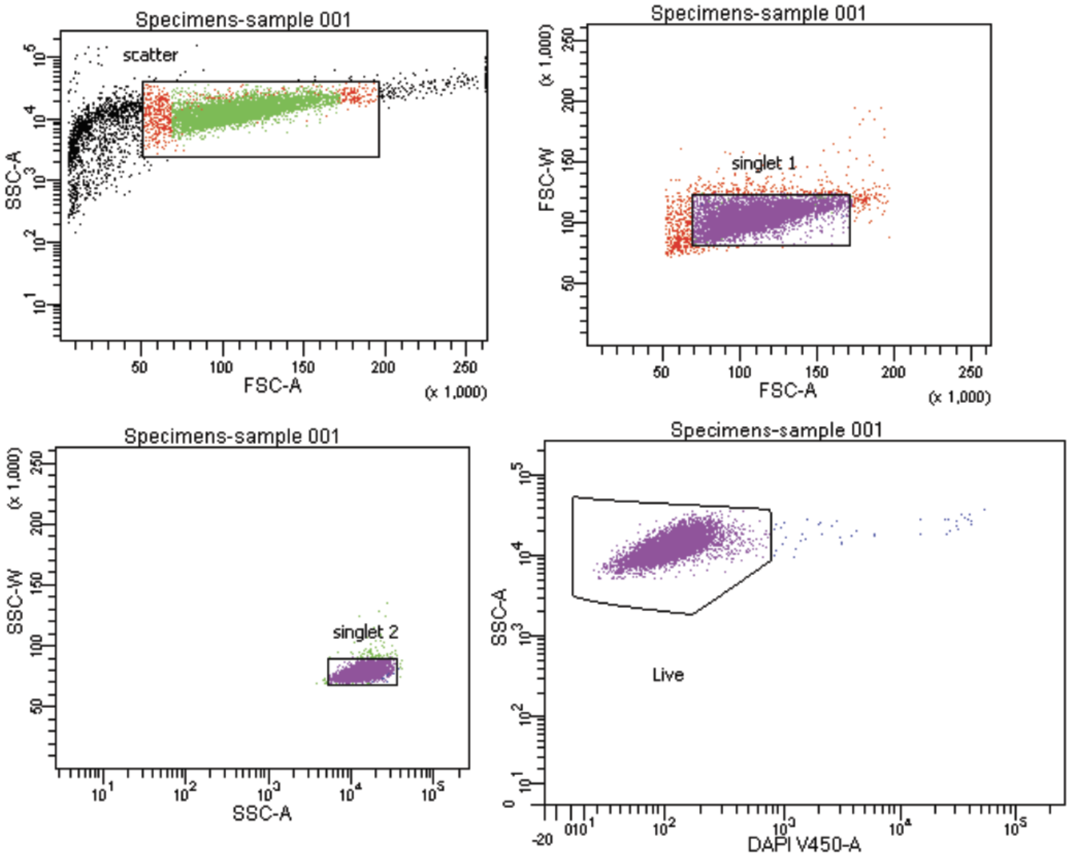

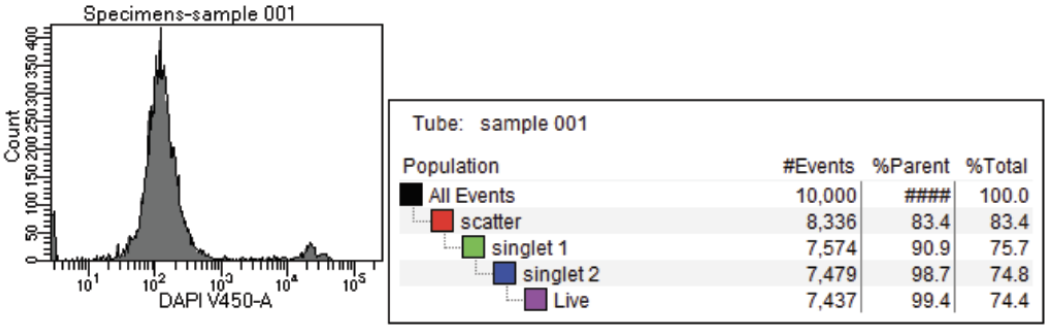

Tube: sample 001

Population	#Events	%Parent	%Total
All Events	10,000	####	100.0
scatter	8,336	83.4	83.4
singlet 1	7,574	90.9	75.7
singlet 2	7,479	98.7	74.8
Live	7,437	99.4	74.4

Fig. S3: Gating strategy for viable singlet T-cells

bioRxiv preprint doi: https://doi.org/10.1101/2025.09.25.678665; this version posted September 25, 2025. The copyright holder for this preprint (which was not certified by peer review) is the author/funder, who has granted bioRxiv a license to display the preprint in perpetuity. It is made available under aCC-BY 4.0 International license.

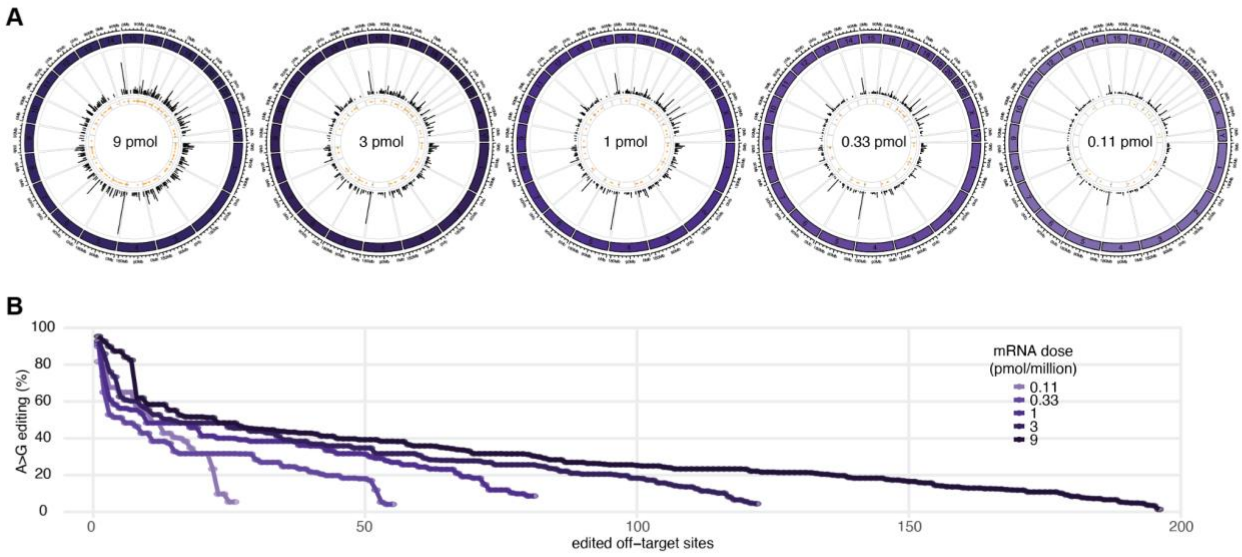

Fig. S4: ABE8e off-target sites at different mRNA doses (pmol/million T cells). A) 339 R-loops (black lines) and edited sites (yellow dots) genome-wide. **B)** Editing frequency at sites within each off-target R-loops.

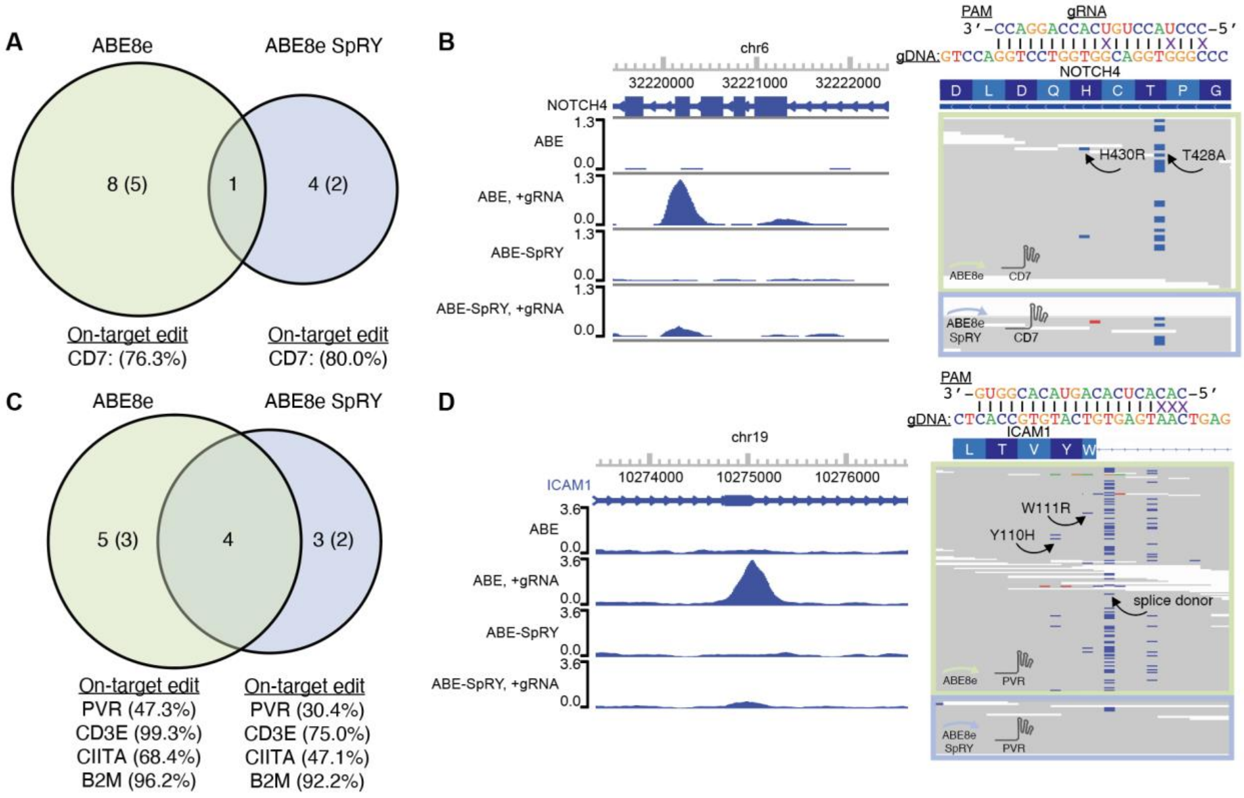

Fig. S5: Off-target sites for therapeutic targets in primary human T-cells. A) Off-target R-loops for CD7 splice site targeting gRNA. The number of edited R-loops is shown in parentheses. **B)** Example off-target site in NOTCH4 tumor-suppressor/oncogene. **C)** Off-target R-loops for quadruple edited T-cells. The number of edited R-loops is shown in parentheses. **D)** Example off-target site disrupting ICAM-1 splice donor.

bioRxiv preprint doi: https://doi.org/10.1101/2025.09.25.678665; this version posted September 25, 2025. The copyright holder for this preprint (which was not certified by peer review) is the author/funder, who has granted bioRxiv a license to display the preprint in perpetuity. It is made available under aCC-BY 4.0 International license.

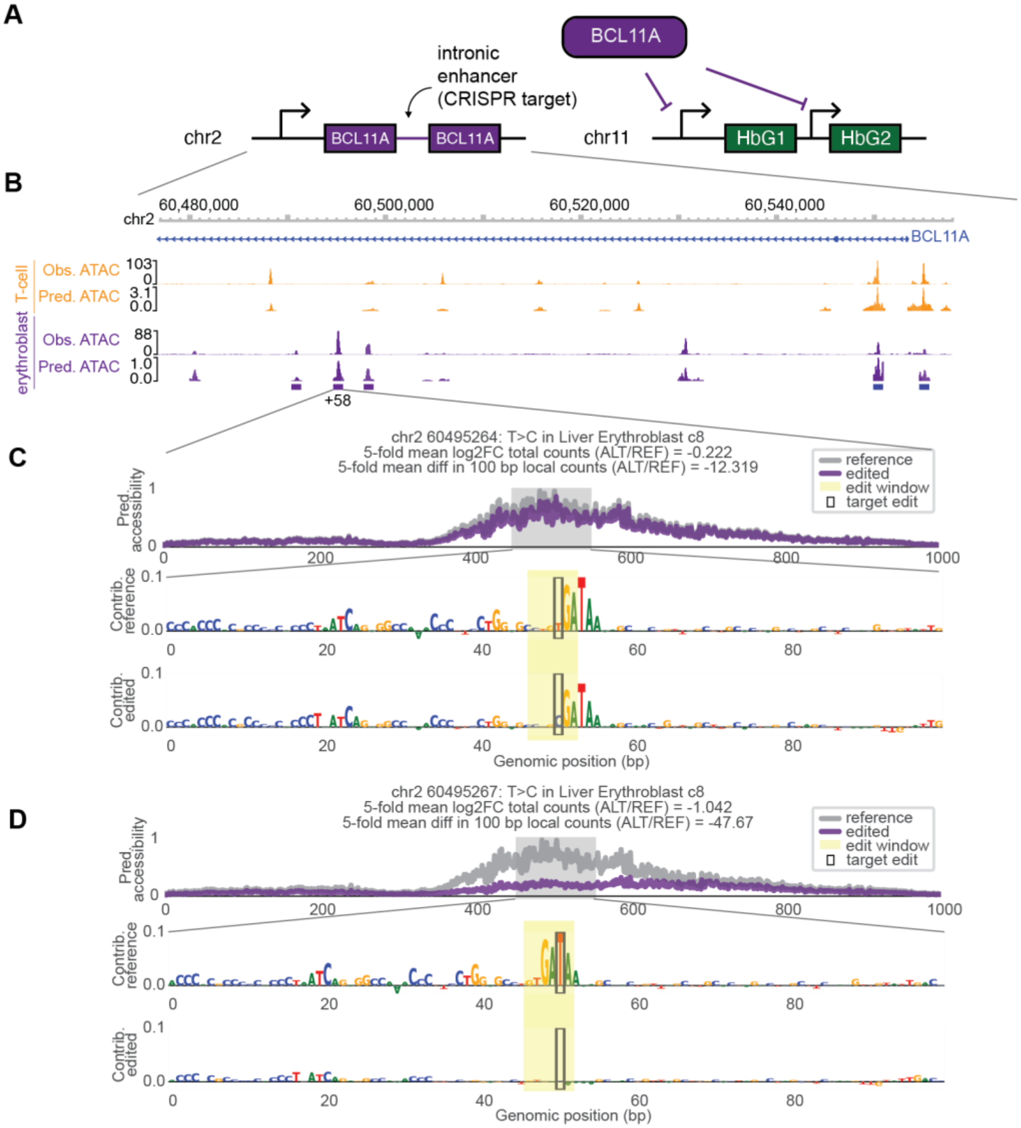

Fig. S6: Erythroblast ChromBPNet model predicts impact of known therapeutic CRISPR target (exagamglogene autotemcel, Casgevy) on accessibility A) Schematic of Casgevy mechanism. BCL11A represses fetal hemoglobin in adulthood. Disruption of a BCL11A enhancer reactivates fetal hemoglobin. **B)** Observed ATAC and predicted accessibility from ChromBPNet BCL11A intron, for T-cells (this study) and erythroblasts (*48*). **C)** Predicted accessibility for chr2 60495264: T>C edit and reference sequence in erythroblasts, along with DeepLIFT contribution scores. **D)** Predicted accessibility of chr2 60495267: T>C edit and reference sequence in erythroblasts. The ABE8e edit window is derived from the highest efficiency gRNA sg1620 (*51*).

bioRxiv preprint doi: https://doi.org/10.1101/2025.09.25.678665; this version posted September 25, 2025. The copyright holder for this preprint (which was not certified by peer review) is the author/funder, who has granted bioRxiv a license to display the preprint in perpetuity. It is made available under aCC-BY 4.0 International license.

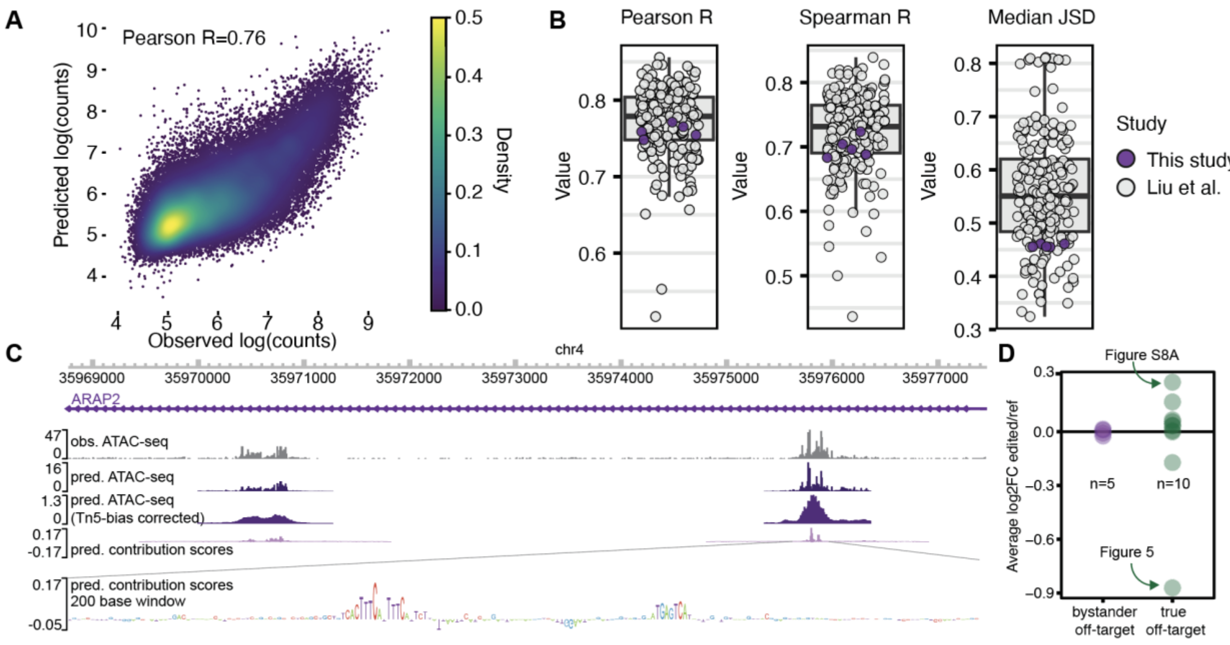

Fig. S7: ChromBPNet model trained on primary human T-cells accurately predicts chromatin accessibility. A) Correlation of observed ATAC-seq log fragment counts in and predicted log counts from ChromBPNet, in peak regions, for one model fold. **B)** Performance of 5-fold models in this study compared to 189 models from prior study (*48*). Higher R and Lower Jensen-Shannon Distances (JSD) are more favorable. **C)** Example browser track showing observed accessibility (ATAC-seq Tn5 insertions), predicted accessibility from ChromBPNet, and Tn5 bias-corrected predictions from ChromBPNet. **D)** Log2 fold-changes of ChromBPNet-predicted accessibility for edited vs reference sequences by *in silico* mutagenesis, averaged over 5-fold models. Each point represents a unique off-target edit, and edits are stratified into bystander edits (occurring within the on-target R-loop) and true off-targets (occurring in an off-target R-loop).

bioRxiv preprint doi: https://doi.org/10.1101/2025.09.25.678665; this version posted September 25, 2025. The copyright holder for this preprint (which was not certified by peer review) is the author/funder, who has granted bioRxiv a license to display the preprint in perpetuity. It is made available under aCC-BY 4.0 International license.

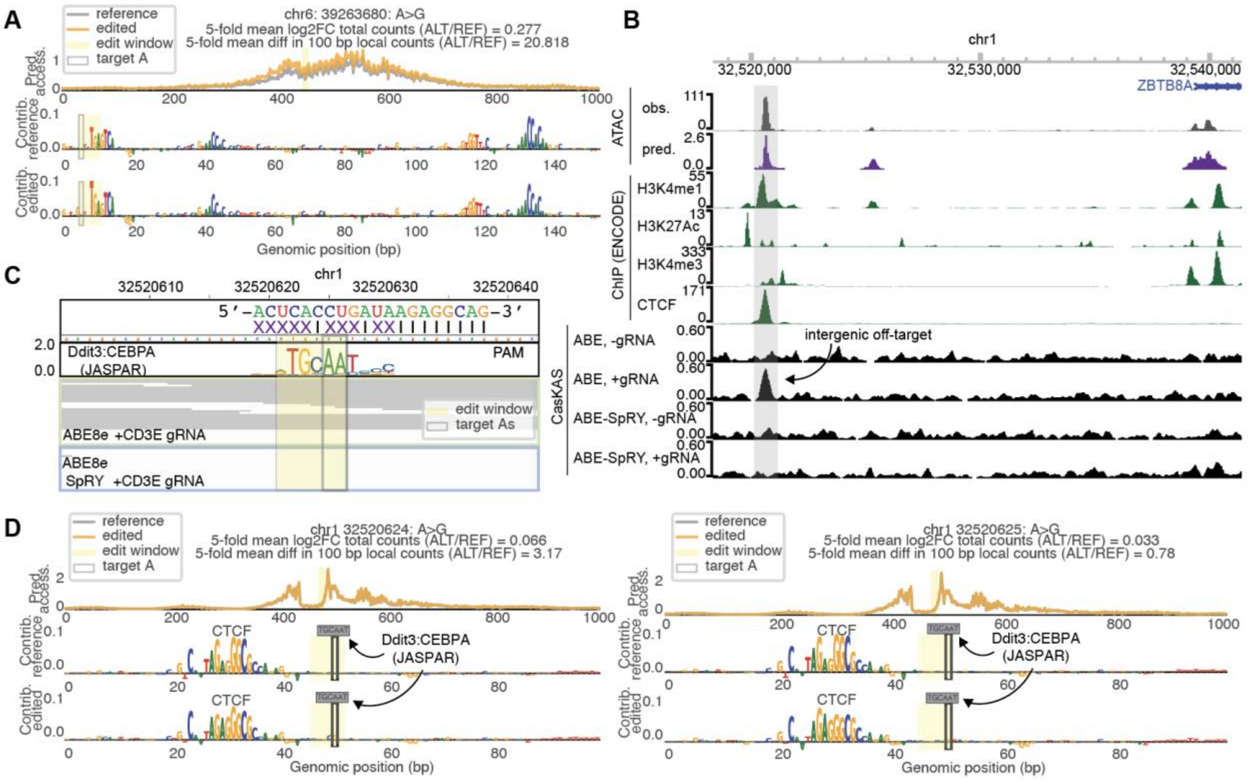

Fig. S8: Additional ChromBPNet *in silico* mutagenesis examples A) Predicted effect of chr6: 39263680: A>G mutation on accessibility. **B)** Off-target ABE8e + CD3E gRNA site overlapping with ATAC, H3K4me1 ChIP-seq, and CTCF ChIP-seq. **C)** Putative gRNA binding site with eight consecutive seed matches (black lines) and canonical GGG PAM. PWM for JASPAR-identified motif based on *in vitro* SELEX data. **D)** Predicted effect of chr1 32520624: A>G and chr1 32520625: A>G mutations on accessibility. The neighboring motif was identified as a CTCF motif by TOMTOM (p-value 2.49x10^{-4}).

bioRxiv preprint doi: https://doi.org/10.1101/2025.09.25.678665; this version posted September 25, 2025. The copyright holder for this preprint (which was not certified by peer review) is the author/funder, who has granted bioRxiv a license to display the preprint in perpetuity. It is made available under aCC-BY 4.0 International license.

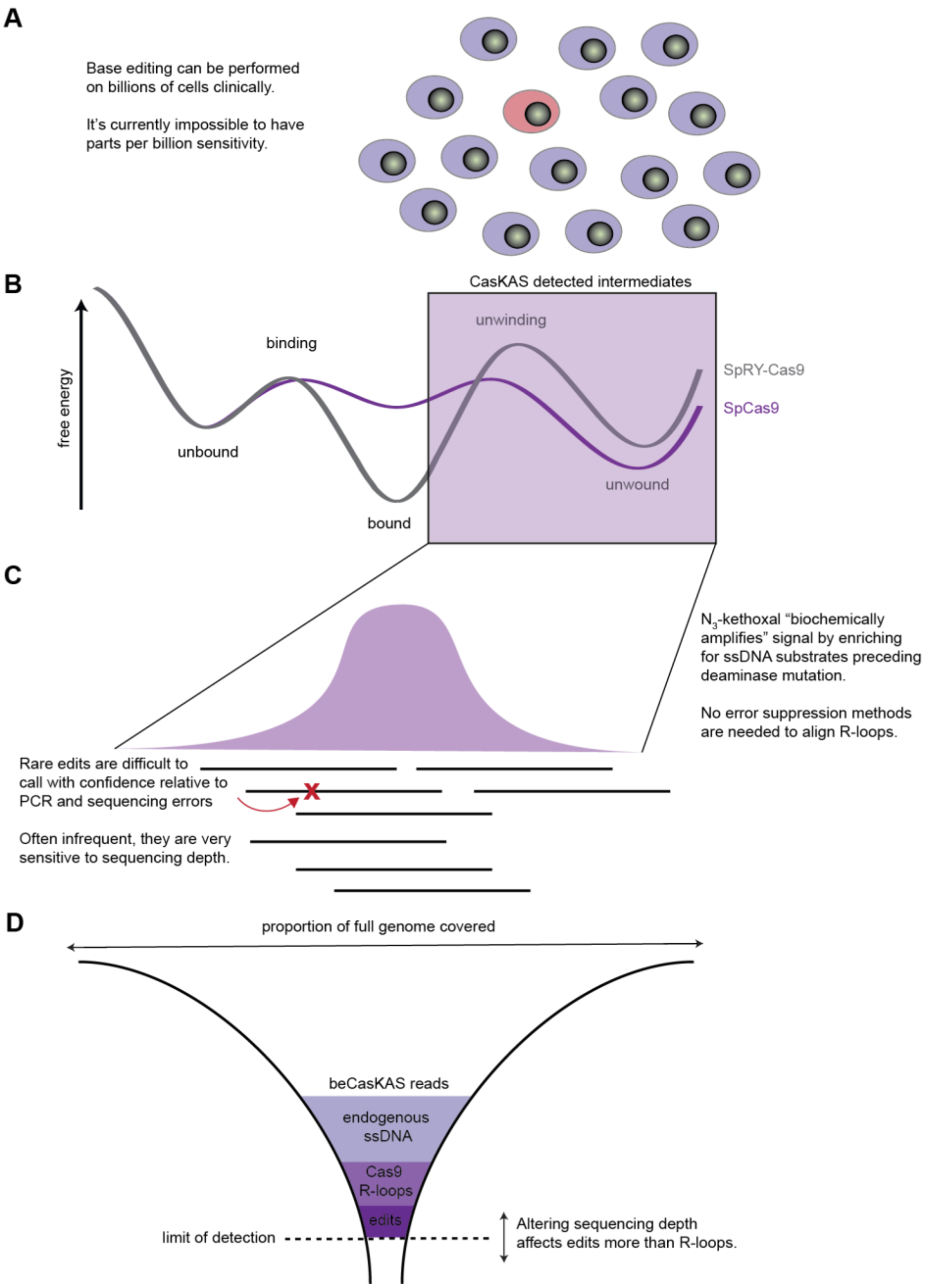

Fig. S9: Signal amplification in beCasKAS. A) Scale of common clinical base editing workflows. **B)** Cartoon free energy diagram of SpCas9 and SpRY-Cas9 adapted from ref (*43*). **C)** Example gRNA-dependent beCasKAS peak. **D)** Breakdown of reads sequenced by beCasKAS.

bioRxiv preprint doi: https://doi.org/10.1101/2025.09.25.678665; this version posted September 25, 2025. The copyright holder for this preprint (which was not certified by peer review) is the author/funder, who has granted bioRxiv a license to display the preprint in perpetuity. It is made available under aCC-BY 4.0 International license.

Table S1: Primers and PCR conditions

Primer Name	Sequence	Purpose
Q5_hA3A_F130Y_F	TGCCCGCATCtatGATTACGACC	Generate WT hA3A
Q5_hA3A_F130Y_R	GCGAAGATACGCAGTCTCAC	Generate WT hA3A
98C for 1 min, 25x [98C for 10s, 67C for 30s, 72C for 4 min], 72C for 5 min, 4C hold		
Q5_HEK4_F	GGCACTGCGGCTGGAGGTGGGTTTTAGAGCTAGAAATAGCAAGTTAAAATAAGGC	Clone HEK4 gRNA
Q5_gRNA_R	GGTGTTTCGTCCTTTCCACAAG	Clone any gRNA
98C for 1 min, 25x [98C for 10s, 66C for 30s, 72C for 2 min], 72C for 5 min, 4C hold		
Q5_ABE8e_mRNA_F	CTCAGAGAGAACCCGCCACCATGAAACGGACAGCCGAC	Clone plasmid with 5’UTR, 3’UTR, and polyA tail
Q5_ABE8e_mRNA_R	TAGGCCACCGAGGCTCCAGCTTAGACTTTCCTCTTCTTCTTGG	Clone plasmid with 5’UTR, 3’UTR, and polyA tail
98C for 1 min, 25x [98C for 10s, 62C for 30s, 72C for 4 min], 72C for 5 min, 4C hold		
PolyA-Sanger-F	CCTCGGTGGCCTAGCTTCTT	Sanger Sequencing Primer

Table S2: sgRNAs

sgRNA name	Protospacer sequence (provided as a DNA sequence)
HEK4	GGCACTGCGGCTGGAGGTGG
CD7	CCCTACCTGTCACCAGGACC
PVR	CACACTCACAGTACACGGTG
CD3E	ACTCACCTGATAAGAGGCAG
CIITA	CACTCACCTTAGCCTGAGCA
B2M	ACTCACGCTGGATAGCCTCC

bioRxiv preprint doi: https://doi.org/10.1101/2025.09.25.678665; this version posted September 25, 2025. The copyright holder for this preprint (which was not certified by peer review) is the author/funder, who has granted bioRxiv a license to display the preprint in perpetuity. It is made available under aCC-BY 4.0 International license.

Table S3: ChromBPNet performance metrics

Fold	Spearman correlation (predicted vs observed log counts)	Pearson correlation (predicted vs observed log counts)	Median Jensen-Shannon distance (predicted vs observed profile)	Max Tn5 response
0	0.696	0.759	0.455	0.001
1	0.688	0.754	0.461	0.001
2	0.704	0.765	0.456	0.001
3	0.683	0.748	0.461	0.001
4	0.724	0.771	0.456	0.001
mean	0.699	0.760	0.458	
